# The LuxS/AI-2 quorum sensing system regulates osmotic adaptation, motility, and type VI secretion system in *Halomonas elongata*

**DOI:** 10.3389/fmicb.2026.1850263

**Published:** 2026-06-23

**Authors:** Zhuo Wang, Yanyan Yang, Xin Guo, Chuanxu Wang, Jing Yang, Yajie Niu, Xin Li

**Affiliations:** 1 Shanxi Key Laboratory of Yuncheng Salt Lake Ecological Protection and Resource Utilization, College of Life Sciences, Yuncheng University, Yuncheng, Shanxi, China; 2 College of Food Science and Engineering, Shanxi Agricultural University, Taigu, Shanxi, China; 3 Department of Life Sciences, Xinzhou Normal University, Xinzhou, Shanxi, China

**Keywords:** autoinducer-2, *Halomonas elongata*, LuxS, motility, osmotic adaptation, type VI secretion system

## Abstract

As a widespread quorum sensing (QS) system in bacteria, the LuxS/AI-2 QS system can coordinate bacterial group behaviors to adapt to diverse environments. However, its roles in halophiles remain largely unexplored. Here, we report that the AI-2 activity of *Halomonas elongata*, a moderately halophilic bacterium, is closely associated with environmental salinity. Moreover, *luxS* deletion abolishes AI-2 production and impairs the growth of *H*. *elongata* at various salt concentrations. Transcriptomic and qRT-PCR analyses revealed that the LuxS/AI-2 QS system regulates the transcription of the compatible solute ectoine degradation genes *doeCD*, thereby modulating intracellular ectoine levels to facilitate osmotic adaptation. Furthermore, the LuxS/AI-2 QS system is also involved in motility and the type VI secretion system (T6SS) in *H*. *elongata*. This work provides new insights into the regulatory mechanism of LuxS/AI-2 QS system underlying osmoadaptation in halophiles.

## Introduction

Halophiles are a group of microorganisms that thrive in environments with high salt concentrations, such as salt lakes, saline soils, and salt mines (Yin et al., 2015; Shu and Huang, 2022; de Souza Vieira and Totola, 2025). Based on their optimal salt concentration for growth, they are generally divided into three groups: slight halophiles (0.2–0.5 M or 1–3% NaCl), moderate halophiles (0.5–2.5 M or 3–15% NaCl), and extreme halophiles (2.5–5.2 M or 15–30% NaCl; Kushner, 1978). *Halomonas elongata* is a moderately halophilic, Gram-negative bacterium isolated from a solar saltern (Vreeland et al., 1980; Schwibbert et al., 2011). It is a well-established model organism for studying the molecular basis of bacterial osmoadaptation, as it grows at a wide range of salinities (0.5–3.0 M NaCl; Vreeland et al., 1980; Gunde-Cimerman et al., 2018; Hobmeier et al., 2022). Moreover, *H*. *elongata* has significant potential for industrial applications, producing various bioactive metabolites (Yin et al., 2015; Benitez-Mateos and Paradisi, 2023; Yan et al., 2025).

A critical osmoadaptation strategy that enables halophiles to cope with hypersaline environments is the synthesis and accumulation of compatible solutes, which are highly water-soluble organic molecules that do not impair cell metabolism even at high intracellular levels (Gunde-Cimerman et al., 2018; Shu and Huang, 2022). *H*. *elongata* achieves a broad salt tolerance by accumulating the compatible solute ectoine in the cytoplasm (Schwibbert et al., 2011; Kindzierski et al., 2017). The metabolic pathway of ectoine has been well-characterized in *H*. *elongata*. LysC, an aspartate kinase, converts L-aspartic acid to L-4-aspartyl-phosphate, which is then converted by Asd to L-aspartate-4-semialdehyde, a key intermediate in the aspartate pathway (Schwibbert et al., 2011; Hermann et al., 2020). Subsequently, three enzymes encoded by the conserved *ectABC* operon are involved in ectoine *de novo* synthesis (Hu et al., 2024; Zou et al., 2026). The L-2,4-diaminobutyric acid (DABA) transaminase EctB converts L-aspartate-4-semialdehyde to DABA, which is acetylated by EctA (DABA-Nγ-acetyltransferase) to form Nγ-acetyl-L-2,4-diaminobutyric acid (Schwibbert et al., 2011). Finally, the cyclic condensation reaction is catalyzed by EctC (ectoine synthase) to yield ectoine. In addition to serving as a compatible solute, ectoine can also be used as a nutrient (Schwibbert et al., 2011). The gene cluster *doeABCD* encodes four enzymes involved in ectoine degradation (Schwibbert et al., 2011; Hermann et al., 2020; Reshetnikov et al., 2020). DoeA (ectoine hydrolase) hydrolyzes ectoine to Nα-acetyl-L-2,4-diaminobutyrate, which is deacetylated by DoeB to form DABA (Schwibbert et al., 2011; Reshetnikov et al., 2020). Then DoeD converts DABA to L-aspartate-4-semialdehyde, which is oxidized by DoeC to aspartate (Schwibbert et al., 2011; Reshetnikov et al., 2020). It has been shown that the promoter of *ectA* contains binding sites for sigma factor σ^70^ and σ^38^, while a σ^54^-controlled promoter is located upstream of *ectC* (Schwibbert et al., 2011; Stiller et al., 2018). The *doeX* gene, adjacent to the *doeAB* operon, encodes an AsnC/Lrp family transcriptional regulator that controls *doeAB* transcription (Schwibbert et al., 2011). However, the *doeCD* genes are not part of the *doeABX* operon and the underlying regulatory mechanism remains to be elucidated.

Quorum sensing (QS) is a complex cell-to-cell communication system that orchestrates bacterial group behaviors in a population density-dependent manner (Papenfort and Bassler, 2016; Fan et al., 2022). It is mediated by small signaling molecules and plays a pivotal role in bacterial adaptation to their environment (Papenfort and Bassler, 2016; Fan et al., 2022). A variety of QS signal molecules have been identified to date such as autoinducer-2 (AI-2), acyl-homoserine lactones (AHLs), and Pseudomonas quinolone signal (PQS; Papenfort and Bassler, 2016; Lin et al., 2018). LuxS is a bifunctional enzyme that plays a key role in the activated methyl cycle (AMC) and also catalyzes the production of the quorum sensing signal AI-2 from S-ribosylhomocysteine (Pereira et al., 2013). Notably, the AI-2 synthase LuxS is widespread and exists in both Gram-positive and Gram-negative bacteria, thereby mediating intra- and inter-species communication (Pereira et al., 2013). Beyond roles in bioluminescence, virulence, chemotaxis, and biofilm formation, the LuxS/AI-2 QS system has also been demonstrated to enhance bacterial stress resistance under adverse environmental conditions (Wang et al., 2018, 2019; Song and Wood, 2021; Deng et al., 2022; Cao et al., 2023; Zhang et al., 2025). Nevertheless, the characterization of the LuxS/AI-2 QS system in halophilic microorganisms remains unclear.

In the present study, the role of the LuxS/AI-2 QS system in *H*. *elongata* was investigated. Unexpectedly, we found that the AI-2 activity of *H*. *elongata* is stimulated with increasing sodium chloride concentration up to 1.5 M but decreased above this concentration. Functional analysis revealed that the LuxS/AI-2 QS system contributes to osmotic adaptation in *H*. *elongata* by regulating the transcription of the *doeCD* genes, thereby modulating intracellular ectoine levels. Furthermore, the LuxS/AI-2 QS system is also involved in motility and the type VI secretion system (T6SS) in *H*. *elongata*. These findings may help further our understanding of the regulatory mechanism of the LuxS/AI-2 QS in *Halomonas* and other bacteria.

## Materials and methods

### Bacterial strains and growth conditions

The bacterial strains and plasmids used in this study are listed in Supplementary Table S1. *H*. *elongata* DSM 2581 and derivatives were grown at 30 °C in LB1M medium (tryptone 1%, yeast extract 0.5%, NaCl 1 M). *E*. *coli* strains were cultured at 37 °C in LB medium (tryptone 1%, yeast extract 0.5%, NaCl 0.17 M). *V*. *harveyi* MM32 was grown at 30 °C in AB medium (Bassler et al., 1993). Antibiotics were added at the following concentrations: Kanamycin, 50 μg/ml; Chloramphenicol, 20 μg/ml for *E*. *coli* and Ampicillin, 100 μg/ml; Kanamycin, 50 μg/ml; Chloramphenicol 20 μg/ml for *H*. *elongata*.

### Plasmid construction

Primers used in this study are listed in Supplementary Table S2. The suicide plasmid pK18*mobsacB*-Δ*luxS* was constructed to prepare the Δ*luxS* in-frame deletion mutant. Briefly, the 927-bp upstream fragment and 902-bp downstream fragment of *luxS* were amplified with primer pairs *luxS*UF-*BamH*I/*luxS*UR and *luxS*DF/*luxS*DR-*Sal*I. Then, the upstream and downstream fragments were inserted between *BamH*I and *Sal*I restriction sites of pK18*mobsacB* by Seamless Cloning, resulting in pK18*mobsacB*-Δ*luxS*. To express His-tagged LuxS (HELO_2343) and Pfs (HELO_2341), primers *luxS*F-*BamH*I/*luxS*R-*Sal*I and *pfs*F-*BamH*I *pfs*R-*Sal*I were used to amplify *luxS* and *pfs* fragments from genomic DNA of *H*. *elongata*. The PCR products of *luxS* and *pfs* were inserted into BamHI and *Sal*I restriction sites of pET28a by Seamless Cloning, resulting in plasmids pET28a-*luxS* and pET28a-*pfs*. To complement the *luxS* mutant, primers *luxS*F-*Kpn*I and *luxS*R-*Hind*III were used to amplify the *luxS* gene fragment from *H*. *elongata* genomic DNA. The PCR product of *luxS* was inserted into the KpnI/HindIII sites of pBBR1MCS1 by Seamless Cloning, resulting in plasmid pBBR1MCS1-*luxS*. pBBR1MCS2-*hcp*-*his*_6_ was constructed in the same way by using the primers listed in Supplementary Table S2. To construct the *sfGFP* fusion reporter vector P*luxS*-*sfGFP*, primers P*luxS*F-*EcoR*I and P*luxS*R-*Sal*I were used to amplify the *luxS* promoter region from *H*. *elongata* genomic DNA. The PCR product of the *luxS* promoter region was inserted into the *EcoR*I/*Sal*I sites of pKHGZ by Seamless Cloning, resulting in plasmid P*luxS*-*sfGFP*. The *sfGFP* fusion reporter vectors P*ectA*-*sfGFP*, P*doeC*-*sfGFP*, P*fliF*-*sfGFP*, and P*t6ss*-*sfGFP* were constructed in similar manners by using the primers listed in Supplementary Table S2. The integrity of the insert in all constructs was confirmed by Sanger sequencing.

### In-frame deletion and complementation in *H*. *elongata*

To construct in-frame deletion mutants, pK18*mobsacB* derivatives were transformed into relevant *H*. *elongata* strains through *E*. *coli* S17-1(λpir)-mediated conjugation (Grammann et al., 2002), and the transconjugants were selected by plating on LB1M agar plates supplemented with Ampicillin (100 μg/ml) and kanamycin (50 μg/ml). The in-frame deletion mutants were subsequently screened on LB1M plates containing 20% sucrose. All mutants were verified by PCR and DNA sequencing. For complementation, the pBBR1MCS1-*luxS* plasmid was transformed into Δ*luxS* mutant through *E*. *coli* S17-1(λpir)-mediated conjugation, and the transconjugants were selected by plating on LB1M agar plates containing kanamycin (50 μg/ml).

### Protein expression and purification

The His_6_-tagged protein was expressed in *E*. *coli* strain BL21(DE3). For protein production, bacteria cultures were cultivated in LB medium at 37 °C until OD_600_ reached 0.5, then shifted to 22 °C and induced with 0.3 mM isopropyl β-D-1-thiogalactopyranoside (IPTG) for an additional 12 h. Harvested cells were washed and resuspended in His binding buffer, and lysed by sonication. The His_6_-tagged proteins were purified with the His·Bind Ni-NTA resin (Novagen) according to the manufacturer's instructions. Purified proteins were dialyzed overnight at 4 °C and stored at −80 °C until used.

### *In vitro* synthesis of AI-2 and bioluminescence assays

*In vitro* synthesis of AI-2 was performed as previously described (Zhang et al., 2020). In brief, purified LuxS protein (1 mg/ml) and Pfs protein (1 mg/ml) were added to a reaction buffer (50 mM Tris–HCl, pH 7.5, 500 mM NaCl) containing 1 mM S-adenosylhomocysteine and incubated at 30 °C for 1 h. After incubation, the mixture was filtered through Amicon Ultra-4 filters (limited 3000-molecular-weight cutoff; Millipore) to remove proteins. AI-2 levels in the filtrate were measured using Ellman's reagent (DTNB; Plummer et al., 2011). For exogenous AI-2 supplementation, the synthesized AI-2 was dissolved in sterile water to 1 mM and added to the Δ*luxS* mutant culture at a final concentration of 1 μM at the time of inoculation. An equal volume of sterile water was added to the control group. For bioluminescence assay, an overnight culture of the *V*. *harveyi* MM32 reporter strain in AB medium was diluted 1:5000 into fresh medium (Zhang et al., 2025). Then, 90 μl aliquots of the diluted cells were added to a white, flat-bottomed 96-well microtiter plate (Corning, USA). *H*. *elongata* strains were grown in LB medium supplemented with various concentrations of NaCl at 30 °C with shaking at 220 rpm until the culture reached an OD_600_ of 1.2. The culture was then centrifuged at 8000 rpm for 10 min at 4 °C to remove bacterial cells. The resulting supernatant was carefully collected and filtered through a 0.22 μm filter (Millipore). A volume of 10 μl of the cell-free supernatant was added to the wells, and the plate was incubated at 30 °C for 6 h with shaking at 150 rpm. Luminescence was detected using a chemiluminescence imaging system (Tanon 4600, China). Quantification of luminescence intensity was measured using the BioTek FLx800 Microplate Reader. AI-2 activity is reported as fold induction relative to the buffer control.

### Measurement of the promoter activities by *sfGFP* fusions

Expression of the *sfGFP*-based reporters was measured as previously described (Boas Lichty et al., 2023; Zou et al., 2024). The *sfGFP* reporters were transformed into relevant *H*. *elongata* strains through *E*. *coli* S17-1(λpir)-mediated conjugation. The resulting strains were grown overnight in LB1M medium. The cultures were then diluted 1:100 into fresh LB1M medium and grown to an OD_600_ of 1.2. Subsequently, 200 μl of each culture was transferred to a black-walled, clear-bottom 96-well plate. Fluorescence (excitation/emission: 488/510 nm) was measured using the BioTek FLx800 Microplate Reader, and bacterial growth was monitored (OD_600_) with the BioTek Epoch 2 plate reader. Specific fluorescence is expressed as relative fluorescence intensity (RFU) divided by the optical density of each well.

### RNA-seq and data analysis

The *H*. *elongata* wild-type and Δ*luxS* mutant strains were cultured in LB1M medium at 30 °C with shaking at 220 rpm until OD_600_ reached 1.2. Total RNA was extracted from each sample by using the TRIzol-based method (Tiangen, China). cDNA libraries were constructed using a SuperScript III reagent kit (Invitrogen Life Technologies) and sequenced by Shanghai Sangon Biotechnology Co., Ltd (Illumina HiSeq 2500). RNA-seq reads were mapped to the *H*. *elongata* DSM 2581 reference genome (ASM19687v2) using Bowtie 2. Differentially expressed genes (DEGs) were identified using DESeq2 (Benjamini–Hochberg adjusted *P* < 0.05 and |log 2 fold change| > 1) for the comparisons between *H*. *elongata* DSM 2581 wild-type and Δ*luxS*. The identified DEGs were enriched by Kyoto Encyclopedia of Genes and Genomes (KEGG) pathway.

### RNA extraction and quantitative real-time PCR

All strains were cultured in LB1M medium to an OD_600_ of 1.2. Total RNA was extracted from each sample by using the TRIzol-based method (Tiangen, China). cDNA was synthesized using PrimeScript Reverse Transcriptase (TaKaRa, China). Quantitative RT-PCR (qRT-PCR) was performed using TransStart green qPCR SuperMix (TransGen Biotech, China) on the CFX Duet Real-Time PCR System (Bio-Rad, USA). The 16S rRNA was used as the internal reference gene.

### Quantification of intracellular ectoine levels

For quantification of intracellular ectoine, *H*. *elongata* strains were grown in 150 ml LB1M at 30 °C to an OD_600_ of 1.2. A total of 50 ml culture was centrifuged at 8,000 rpm for 10 min. The cell pellet was resuspended in 30 ml ultrapure water and lysed by sonication. After centrifugation at 12,000 rpm for 10 min, the supernatant was filtered through a 0.22 μm filter membrane for high-performance liquid chromatography (HPLC) analysis (Zou et al., 2026). Samples were analyzed using an Agilent 1260 Infinity II HPLC system equipped with a C18 reverse-phase column and a UV detector. Ectoine was eluted with 5% phase A (methanol) and 95% phase B (20 mM KH_2_PO4, 7 mM sodium 1-pentanesulfonate, pH 3.0), and the flow rate was 0.8 ml/min. The wavelength of the UV detector was 210 nm. Ectoine (number 81619; Sigma) was purchased from Sigma and used as a standard. The ectoine concentration of samples was determined from a standard curve established with a serially diluted ectoine solution.

### Western blot analysis

Western blot analysis was performed as previously reported (Wang et al., 2015a; Li et al., 2025). Samples were separated by electrophoresis on 12% SDS–PAGE and transferred to polyvinylidene difluoride membranes (Millipore). After blocking with 5% (w/v) bovine serum albumin, the membranes were incubated with the appropriate primary antibodies: anti-His (Solarbio, China) and anti-RNAP (Solarbio, China). After five times washes with TBST buffer (50 mM Tris, 150 mM NaCl, 0.05% Tween 20, pH 7.4), the membranes were incubated with a 1:5,000 dilution of horseradish peroxidase-conjugated secondary antibody (Solarbio, China). Protein signals were detected using the ECL kit following the manufacturer's protocol.

### Swimming motility assay

Swimming motility assay was performed as previously described (Wang et al., 2022). Briefly, *H*. *elongata* strains were cultured overnight in LB1M medium. 2.5 μl of bacterial suspension was injected into the center of the semi-solid agar plates (1 M NaCl, 1% tryptone, and 0.3% Difco Bacto agar). All plates were incubated at 30 °C for 32 h.

### Protein Secretion Assay

Secretion of Hcp was detected according to previously reported (Song et al., 2021). Briefly, *H*. *elongata* strains were grown in 150 ml LB1M at 30 °C to an OD_600_ of 1.2. A 2 ml culture was centrifuged, and the cell pellet was resuspended in 100 μl SDS-loading buffer to prepare the whole-cell lysate sample. For secreted protein collection, 100 ml culture was centrifuged at 8,000 rpm. Subsequently, the culture supernatant was filtered through a 0.22-μm filter (Millipore) to remove residual bacterial cells, and the extracellular proteins were extracted by filtration over a nitrocellulose filter (BA85; Whatman, Germany) three times. Proteins bound to the nitrocellulose filter were eluted by soaking it in 100 μl of SDS sample buffer at 70 °C for 15 min. All sample preparations were normalized based on the culture OD_600_ and the volume processed.

## Results

## AI-2 is synthesized by the LuxS enzyme in *H*. *elongata*

**Figure 1:**
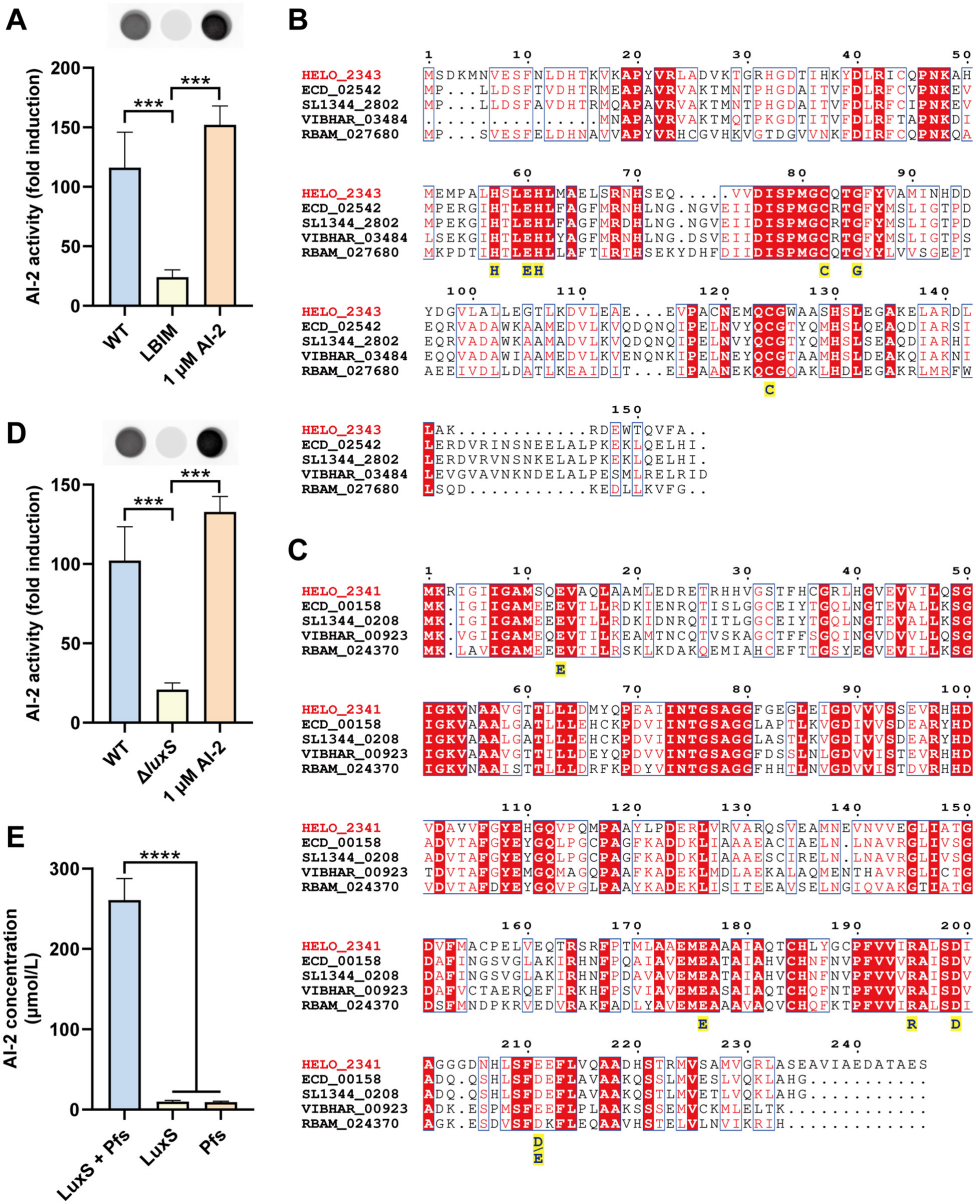
Confirmation of AI-2 activity in *H*. *elongata*. **(A)** AI-2 activity in supernatant of *H*. *elongata* wild-type. Cell supernatants were collected, filtered, and measured for AI-2 activity using the reporter *V*. *harveyi* strain MM32. AI-2 activity is reported as fold induction over the buffer control. Data are shown as mean ± SD from three independent biological replicates (*n* = 3). Statistical significance was determined using one-way ANOVA with Tukey's *post-hoc* test. **(B)** LuxS amino acid multiple sequence alignment. LuxS proteins used in the sequence alignment are as follows: *H*. *elongata* (HELO_2343); *Escherichia coli* (ECD_02542); *Salmonella enterica* subsp. enterica serovar Typhimurium str. SL1344 (SL1344_2802); *Vibrio campbellii* (VIBHAR_03484); *Bacillus velezensis* (RBAM_027680). The important enzyme active sites are shown in blue on a yellow background. **(C)** Pfs amino acid multiple sequence alignment. Pfs proteins used in the sequence alignment are as follows: *H*. *elongata* (HELO_2341); *E*. *coli* (ECD_00158); S. *enterica* serovar Typhimurium SL1344 (SL1344_0208); *V*. *campbellii* (VIBHAR_00923); *B*. *velezensis* (RBAM_024370). The important enzyme active sites are shown in blue on a yellow background. **(D)** AI-2 activity detection of *H*. *elongata* wild-type and Δ*luxS* mutant. Data are shown as mean ± SD from three independent biological replicates (*n* = 3). Statistical significance was determined using one-way ANOVA with Tukey's *post-hoc* test. **(E)** Synthesis detection of AI-2 *in vitro*. The products from the *in vitro* reaction of S-adenosylhomocysteine with LuxS and Pfs were measured using Ellman's reagent. Data are shown as mean ± SD from three independent biological replicates (*n* = 3). Statistical significance was determined using one-way ANOVA with Tukey's *post-hoc* test. ***P < 0.001; ****P < 0.0001.

Although the LuxS/AI-2 QS system is well characterized in *Vibrio harveyi* (Chen et al., 2020) and *Escherichia coli* (Mayer et al., 2023), it remains largely unexplored in halophiles. To test whether *H*. *elongata* produces functional AI-2 molecule, we first determined the ability of the culture supernatant of wild-type to stimulate light production in the AI-2 reporter strain *Vibrio harveyi* MM32 (Miller et al., 2004). The culture supernatant of wild-type promoted light production in strain MM32 at levels comparable to those induced by 1 μM AI-2, whereas the fresh medium did not induce bioluminescence in strain MM32 (Figure 1A). Sequence alignment analysis showed that *H*. *elongata* LuxS and Pfs (S-adenosylhomocysteine nucleosidase) exhibited 40.41–46.15% and 48.48–53.30% sequence identity, respectively, with their counterparts in several other bacteria, including *E*. *coli, Salmonella enterica, Vibrio campbellii*, and *Bacillus velezensis*, and both LuxS and Pfs contain important enzyme active sites (Plummer et al., 2011; Wang et al., 2015b; Figures 1B, C). We further constructed a Δ*luxS* mutant of *H*. *elongata* and confirmed that bioluminescence was abolished in the Δ*luxS* mutant (Figure 1D). In addition, *in vitro* enzymatic assays using S-adenosylhomocysteine as substrate with purified LuxS and Pfs confirmed AI-2 synthesis activity (Figure 1E). These results suggest that AI-2 is produced by LuxS in *H*. *elongata*.

## The LuxS/AI-2 QS system is closely associated with salinity and contributes to osmotic adaptation

**Figure 2:**
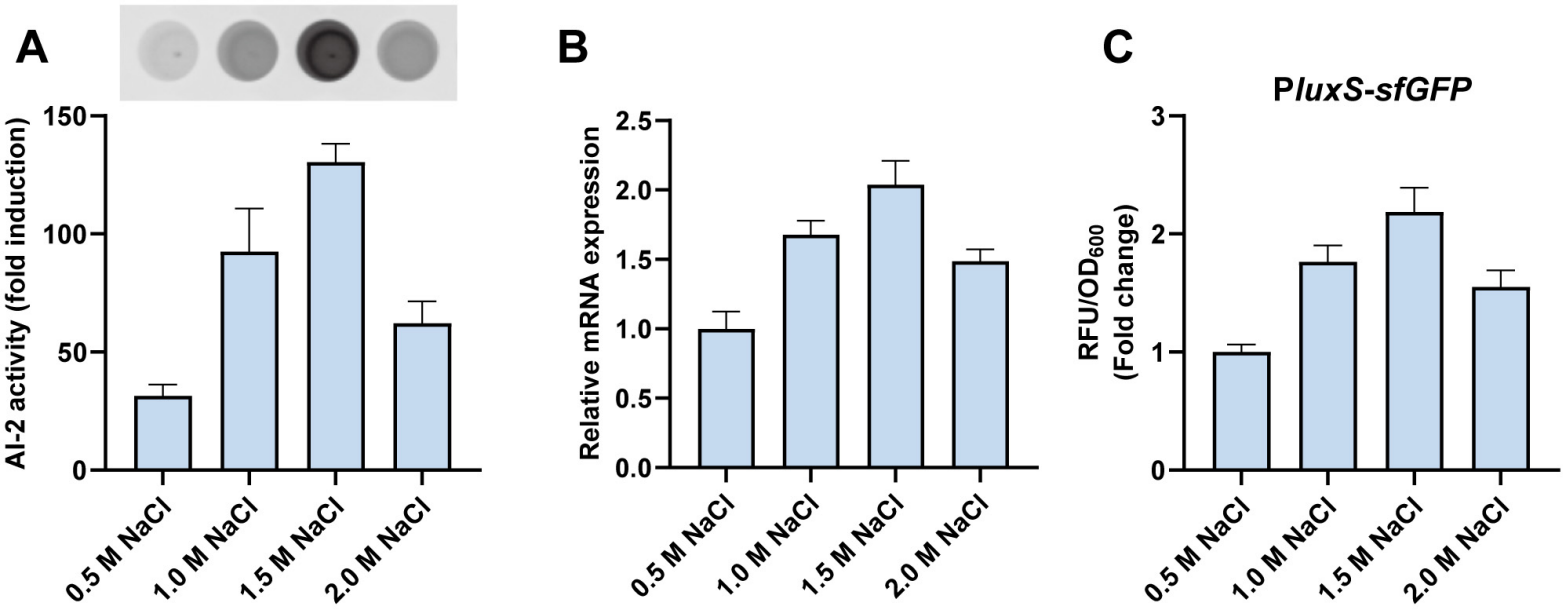
The LuxS/AI-2 QS system is closely associated with salinity. **(A)** AI-2 activity in wild-type *H*. *elongata* at 0.5–2.0 M NaCl. *H*. *elongata* was grown in LB medium supplemented with 0.5, 1.0, 1.5, and 2.0 M NaCl at 30 °C until reaching an OD_600_ of 1.2. Cell-free supernatants were subsequently collected and analyzed for AI-2 activity using the reporter *V*. *harveyi* strain MM32. Data are shown as mean ± SD from three independent biological replicates (*n* = 3). **(B)** The transcription levels of *luxS* at 0.5–2.0 M NaCl in *H*. *elongata*. *H*. *elongata* cells were cultivated under different salinity conditions, and the transcription levels of *luxS* were determined by qRT-PCR. Data are shown as mean ± SD from three independent biological replicates (*n* = 3). **(C)** The promoter activity of *luxS* in wild-type *H*. *elongata* at 0.5–2.0 M NaCl. Cultures were grown at 0.5–2.0 M NaCl and relative fluorescence intensity (RFU) was measured. Data are shown as mean ± SD from three independent biological replicates (*n* = 3).

Several studies have demonstrated that the LuxS/AI-2 QS system promotes bacterial survival in stressful environments (Wang et al., 2019; Cao et al., 2023; Zhang et al., 2025). In general, high-salinity environments are harmful to bacteria, whereas *H*. *elongata* can tolerate salt concentrations well above 1.7 M NaCl (Tanimura et al., 2016). This prompted us to investigate whether the LuxS/AI-2 QS system plays a role in its osmotic adaptation. Interestingly, the AI-2 activity in the culture of wild-type *H*. *elongata* is stimulated by increasing NaCl concentration up to 1.5 M but reduced above this level (Figure 2A). Consistently, quantitative real-time PCR (qRT-PCR) analyses showed that the transcriptional levels of *luxS* exhibited similar trends to those of NaCl concentration (Figure 2B). Promoter activity assays were performed using the *luxS*-*sfGFP* plasmid as the reporter. The results revealed that *luxS* promoter activity was increased with NaCl concentration up to 1.5 M, while declined above this threshold (Figure 2C). These results indicated that the LuxS/AI-2 QS system is closely associated with salinity.

**Figure 3:**
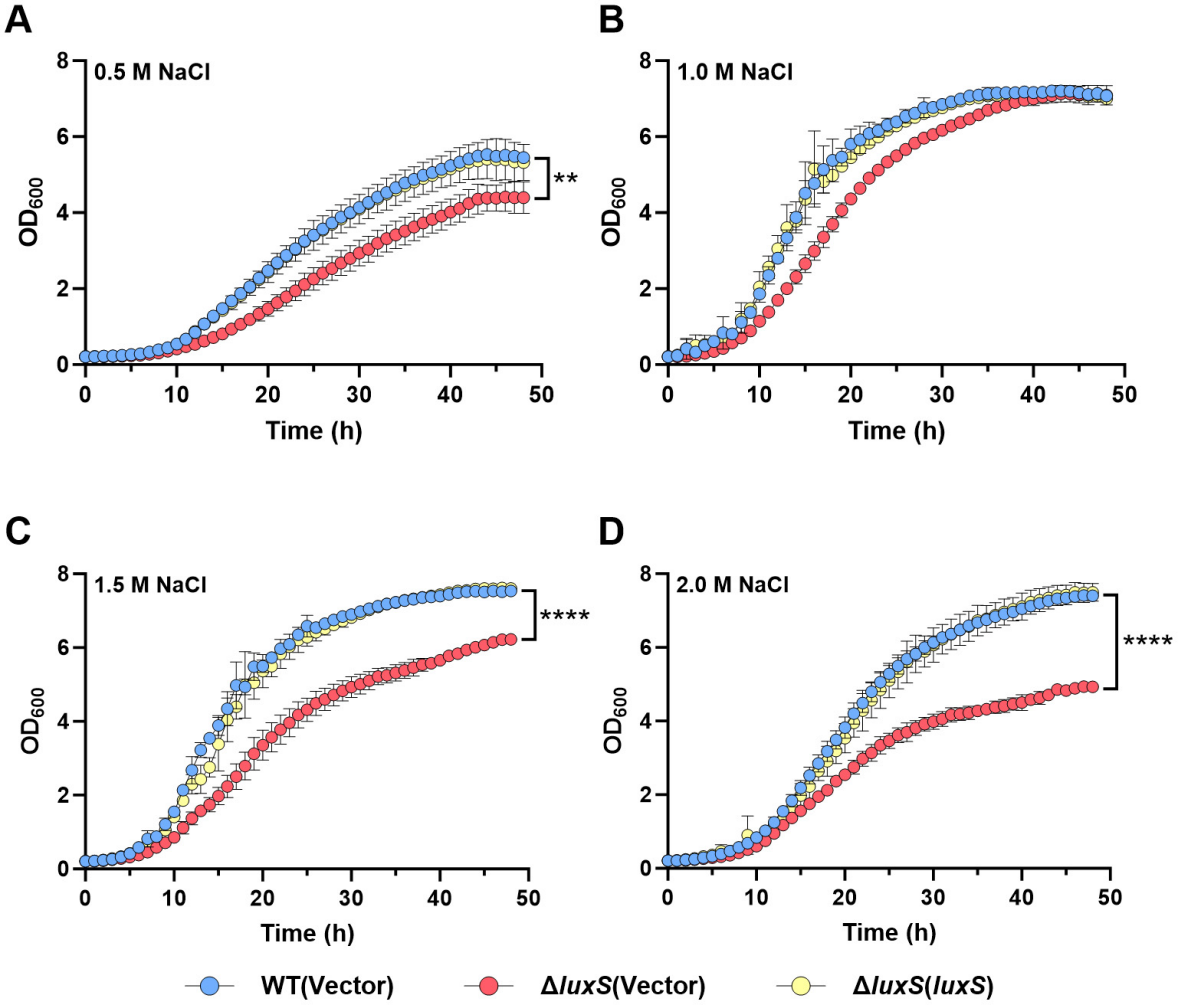
Growth analysis of *H*. *elongata* wild-type, Δ*luxS* mutant, and the complementary strain Δ*luxS* (*luxS*) at various NaCl concentrations. Cells were cultivated in LB medium supplemented with 0.5 M **(A)**, 1.0 M **(B)**, 1.5 M **(C)**, and 2.0 M **(D)** NaCl, and growth was monitored every hour for 48 h at 30 °C with shaking at 300 rpm using the MicroScreen high-throughput real-time microbial growth analysis system (Gering). Statistical significance at the 48 h time point was determined by an unpaired two-tailed Student's *t*-test. Mean and standard deviation of three biological replicates are shown (*n* = 3). **P < 0.01; ****P < 0.0001.

To investigate whether the LuxS/AI-2 QS system contributes to osmotic adaptation in *H*. *elongata*, we monitored the growth of the wild-type, *luxS* mutant, and complemented strains under varying NaCl concentrations. To complement the Δ*luxS* mutant, the pBBR1MCS1 derivative harboring full-length *luxS* under a constitutive *lac* promoter was introduced into the Δ*luxS* mutant via conjugation, and the empty plasmid pBBR1MCS1 served as a vector control in all experiments. It was observed that the absence of the LuxS/AI-2 QS system resulted in severe growth inhibition at 0.5, 1.5, and 2.0 M NaCl compared to the wild-type and complemented strains (*P* < 0.01; Figures 3A, C, D). Additionally, under 1.0 M NaCl, the *luxS* mutant grew slowly during early and mid-exponential phases compared to the wild-type and complemented strains, while no significant difference was observed at stationary phase (Figure 3B). Taken together, these results demonstrated that the LuxS/AI-2 QS system contributes to osmotic adaptation in *H*. *elongata*.

## Transcriptomic analysis of *H*. *elongata* and its *luxS* mutant

To elucidate the molecular mechanism underlying the role of the LuxS/AI-2 QS system in osmotic adaptation of *H*. *elongata*, RNA-seq analysis was performed to compare the transcriptomic profiles of the wild-type and Δ*luxS* mutant strains. Differentially expressed genes (DEGs) between the wild-type and Δ*luxS* mutant strains were identified using DESeq2, with significance defined as an adjusted *P* < 0.05 and |log2 fold change| > 1.

**Figure 4:**
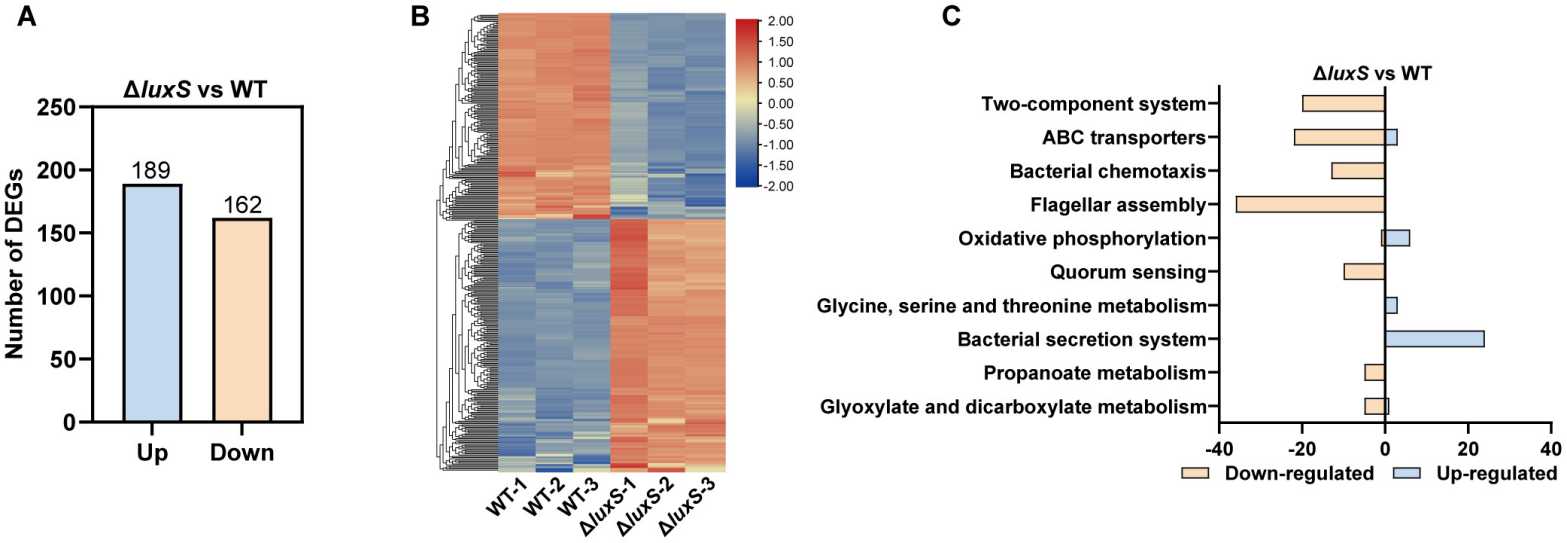
Transcriptome analysis of *H*. *elongata* wild-type and Δ*luxS* strains. **(A)** The number of upregulated and downregulated DEGs in wild-type and Δ*luxS* mutant. **(B)** Cluster heatmap of DEGs at the transcriptional level. **(C)** KEGG pathway enrichment analysis of DEGs between wild-type and Δ*luxS* mutant.

A total of 351 DEGs were identified between the wild-type and Δ*luxS* mutant strains, including 189 upregulated and 162 downregulated genes (Figure 4A and Supplementary Table S3). The heatmap of these DEGs shows consistent expression patterns within each group and differ significantly between wild-type and Δ*luxS* mutant strains (Figure 4B). KEGG pathway enrichment analysis revealed that these DEGs are involved in various processes, including the two-component system, ABC transporters, flagellar assembly, and bacterial secretion system (Figure 4C). Notably, the upregulated transcripts in Δ*luxS* included genes associated with ectoine degradation and T6SS system, such as *doeC, doeD, eutB, vasI, clpV, vipA*, and *vipB* (Supplementary Table S3). In contrast, the repressed transcripts included genes involved in flagellar assembly, such as *flhA, flhB, fliH, flgE, flgD*, and *flgA* (Supplementary Table S3). These results suggest that the LuxS/AI-2 QS system modulates key metabolic and cellular processes in *H*. *elongata*.

## The LuxS/AI-2 QS system modulates intracellular ectoine levels by regulating *doeCD* in *H*. *elongata*

**Figure 5:**
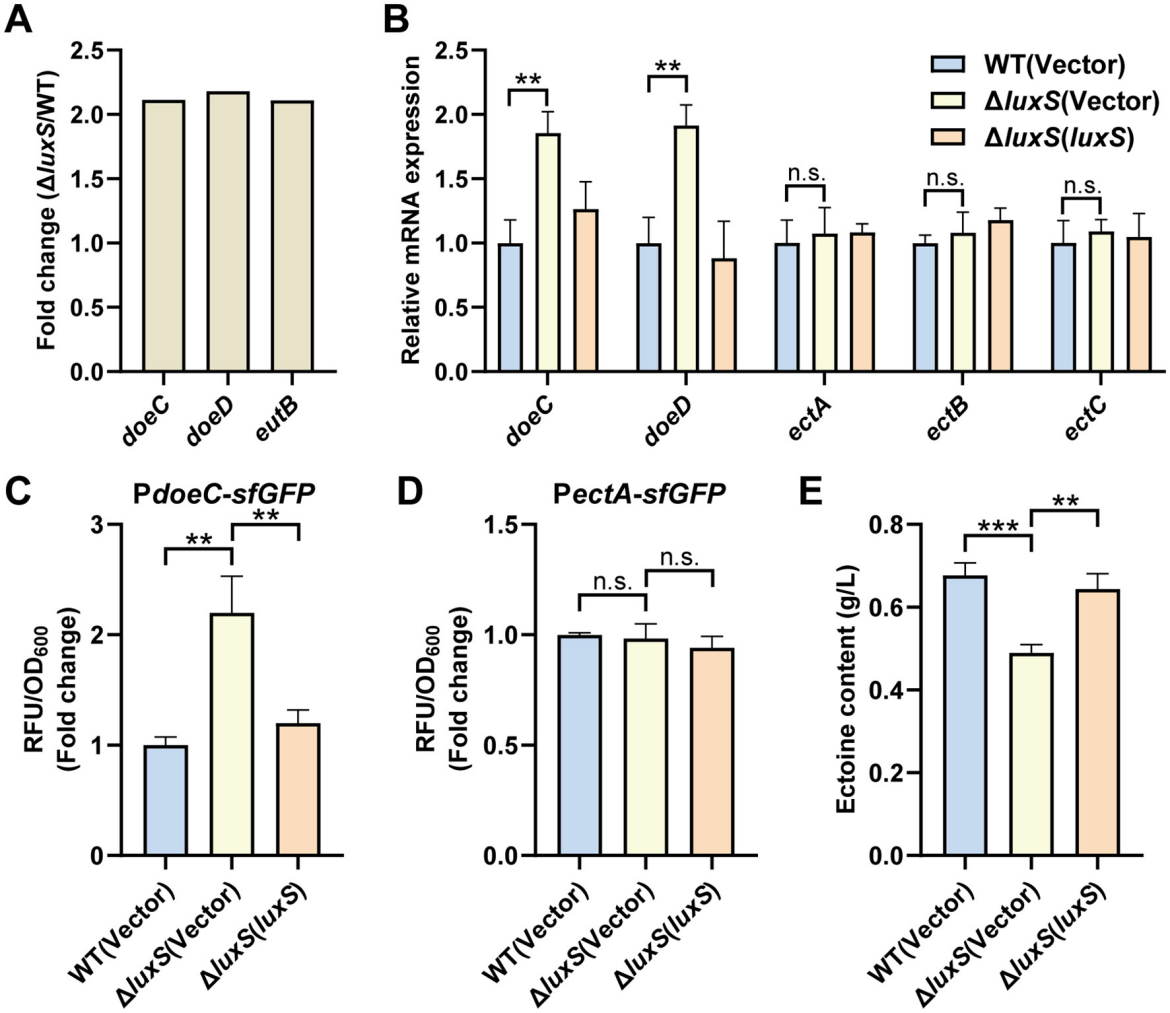
The LuxS/AI-2 QS system controls intracellular ectoine levels by regulating *doeCD*. **(A)** Fold changes of the transcriptional levels of the *doeC, doeD*, and *eutB* genes in the Δ*luxS* mutant vs. wild-type from RNA-seq data. **(B)** The relative mRNA levels of *doeCD* and *ectABC* were measured by qRT-PCR in the wild-type, Δ*luxS* mutant, and complemented strains. Data are shown as mean ± SD from three independent biological replicates (*n* = 3). Statistical significance was determined using one-way ANOVA with Tukey's *post-hoc* test. **(C,D)** The promoter activity of *doeC* (C) and *ectA* (D) in the wild-type, Δ*luxS* mutant, and complemented strains. Data are shown as mean ± SD from three independent biological replicates (*n* = 3). Statistical significance was determined using one-way ANOVA with Tukey's *post-hoc* test. **(E)** The intracellular concentrations of ectoine in the wild-type, Δ*luxS* mutant, and complemented strains were detected by HPLC. Data are shown as mean ± SD from three independent biological replicates (*n* = 3). Statistical significance was determined using one-way ANOVA with Tukey's *post-hoc* test. **P < 0.01; ***P < 0.001; n.s., not significant.

Our RNA-seq data revealed that the deletion of *luxS* induces expression of *doeC, doeD*, and *eutB* genes in *H*. *elongata* (Figure 5A). Previous studies have shown that the enzymes encoded by *doeC* and *doeD*, but not *eutB*, are involved in ectoine degradation (Schwibbert et al., 2011). To validate the transcriptome data, qRT-PCR analysis confirmed that the expression levels of *doeC* and *doeD* were significantly higher in the Δ*luxS* mutant compared to the wild-type and complemented strains (*P* < 0.01; Figure 5B). Interestingly, no significant differences were observed in the expression of the ectoine biosynthesis genes *ectA, ectB*, and *ectC* among the wild-type, Δ*luxS* mutant, and complemented strains (Figure 5B). In addition, the promoter activity assays demonstrated that the transcription of *doeCD* rather than *ectA* was significantly upregulated in the Δ*luxS* mutant (Figures 5C, D). Notably, exogenous AI-2 supplementation inhibited *doeCD* promoter activity to near wild-type levels in the Δ*luxS* mutant (Supplementary Figure S1A). To further examine the role of the LuxS/AI-2 QS system in ectoine metabolism, we measured intracellular ectoine levels in the wild-type, Δ*luxS* mutant, and complemented strains using high-performance liquid chromatography (HPLC). As shown in Figure 5E, the Δ*luxS* mutant exhibited a significant reduction in intracellular ectoine concentration relative to the wild-type and complemented strains (*P* < 0.01). Collectively, these results suggest that the LuxS/AI-2 QS system modulates intracellular ectoine levels by regulating *doeCD* in *H*. *elongata*.

## The LuxS/AI-2 QS system enhances swimming motility in *H*. *elongata*

**Figure 6:**
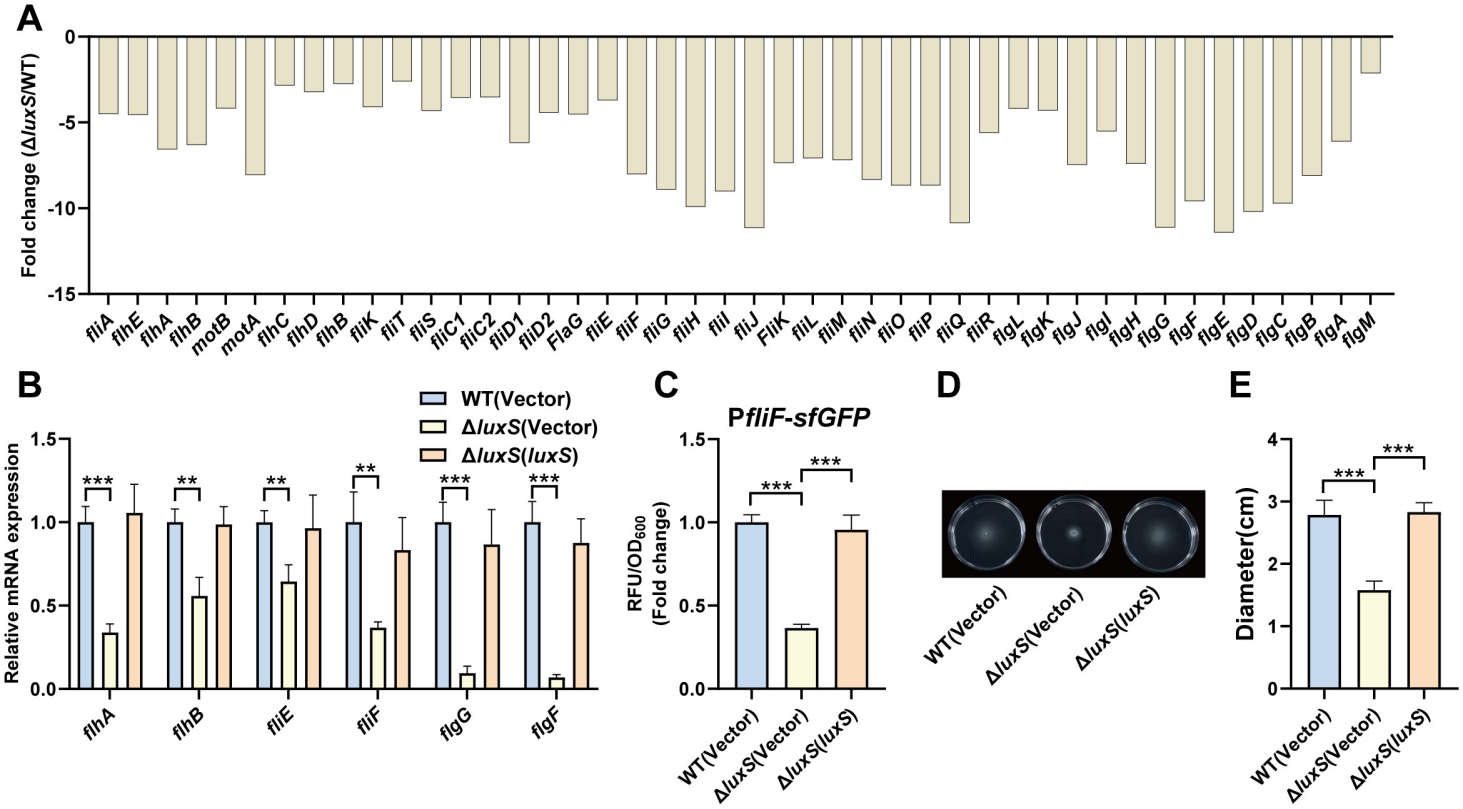
The LuxS/AI-2 QS system enhances swimming motility. **(A)** Fold changes of the transcriptional levels of flagellar genes in the Δ*luxS* mutant vs. wild-type from RNA-seq data. **(B)** The relative mRNA levels of *flhA, flhB, fliE, fliF, flgG*, and *flgF* were measured by qRT-PCR in the wild-type, Δ*luxS* mutant, and complemented strains. Data are shown as mean ± SD from three independent biological replicates (*n* = 3). Statistical significance was determined using two-way ANOVA with Tukey's *post-hoc* test. **(C)** The promoter activity of *fliF* in the wild-type, Δ*luxS* mutant, and complemented strains. Data are shown as mean ± SD from three independent biological replicates (*n* = 3). Statistical significance was determined using one-way ANOVA with Tukey's *post-hoc* test. **(D)** The wild-type, Δ*luxS* mutant, and complemented strains were inoculated on semi-solid plates supplemented with 1.0 M NaCl and incubated at 30 °C for 32 h. **(E)** The swimming motility was assessed by measuring the sizes of swimming zones. Data are shown as mean ± SD from three independent biological replicates (*n* = 3). Statistical significance was determined using one-way ANOVA with Tukey's *post-hoc* test. **P < 0.01; ***P < 0.001.

RNA-seq analysis also showed that the expression levels of almost all flagellar-related genes were significantly downregulated in the Δ*luxS* mutant (Figure 6A and Supplementary Table S3). qRT-PCR analysis confirmed that the expression of flagella assembly genes *flhA, flhB, fliE, fliF, flgG*, and *flgF* was significantly decreased in the Δ*luxS* mutant compared to the wild-type and complemented strains (*P* < 0.01; Figure 6B). The deletion of *luxS* significantly represses the promoter activity of *fliF* compared with the wild-type and complemented strains (*P* < 0.001; Figure 6C). In contrast, exogenous AI-2 supplementation restored swimming motility in the Δ*luxS* mutant (Supplementary Figure S1B). We then sought to investigate whether the LuxS/AI-2 QS system affects motility in *H*. *elongata*. Swimming motility assays were performed by inoculating 2.5 μl of stationary-phase cultures onto the center of semisolid agar plates, and after incubation at 30 °C for 32 h, the swim diameters were measured. The Δ*luxS* mutant exhibited significantly reduced swimming motility compared with the wild-type strain (*P* < 0.001; Figures 6D, E). Expression of *luxS* restored swimming motility in the Δ*luxS* mutant (Figures 6D, E). Altogether, these results suggest that the LuxS/AI-2 QS system promotes swimming motility in *H*. *elongata*.

## The LuxS/AI-2 QS system represses T6SS in *H*. *elongata*

**Figure 7:**
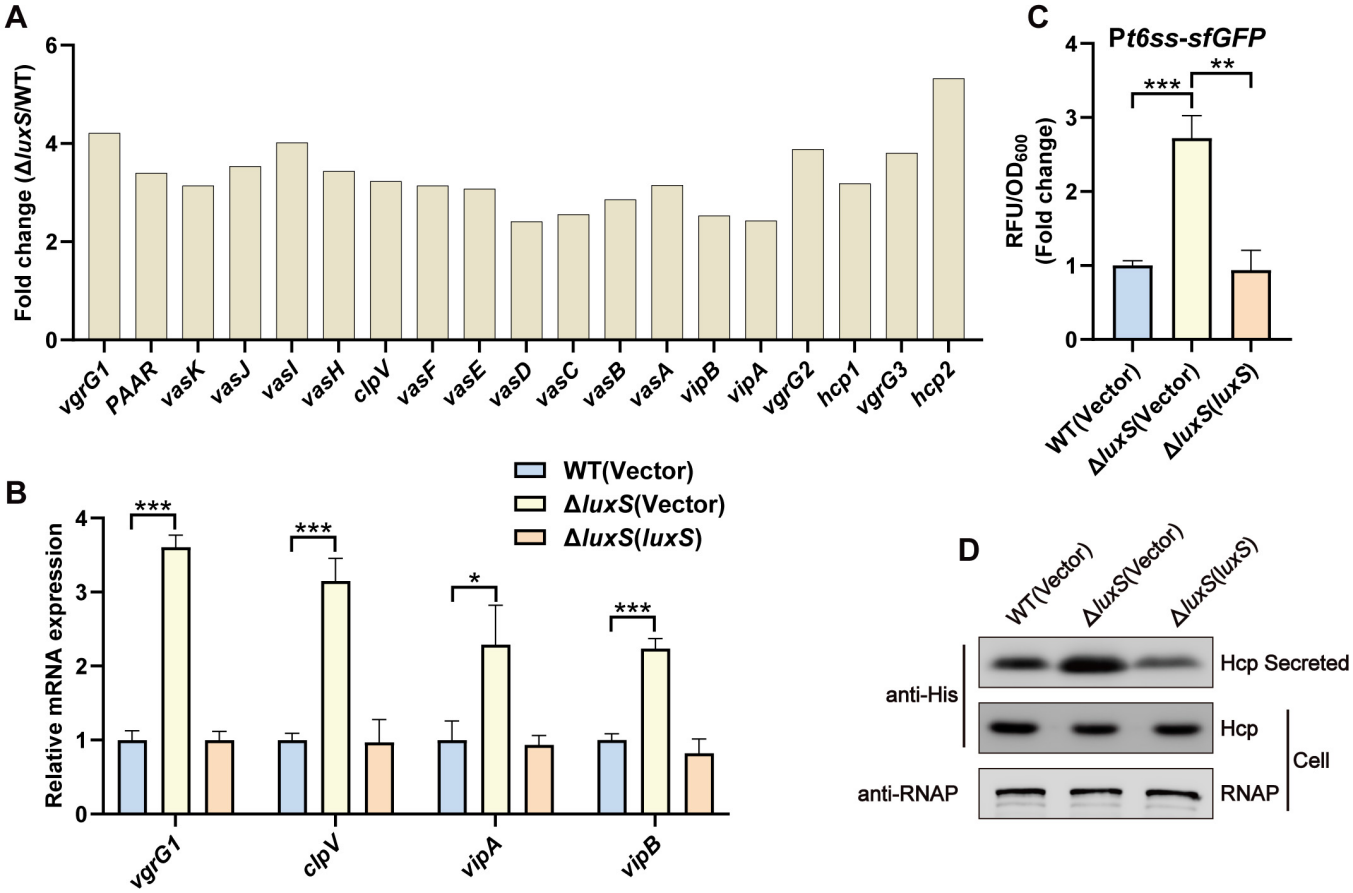
The LuxS/AI-2 QS system represses T6SS. **(A)** Fold changes of the transcriptional levels of T6SS genes in the Δ*luxS* mutant vs. wild-type from RNA-seq data. **(B)** The relative mRNA levels of *vgrG1, clpV, vipA*, and *vipB* were measured by qRT-PCR in the wild-type, Δ*luxS* mutant, and complemented strains. Data are shown as mean ± SD from three independent biological replicates (*n* = 3). Statistical significance was determined using one-way ANOVA with Tukey's *post-hoc* test. **(C)** The promoter activity of *t6ss* in the wild-type, Δ*luxS* mutant, and complemented strains. Data are shown as mean ± SD from three independent biological replicates (*n* = 3). Statistical significance was determined using two-way ANOVA with Tukey's *post-hoc* test. **(D)** Western blot detection of Hcp secretion in *H*. *elongata* wild-type, Δ*luxS* mutant, and complemented strains. His-tagged proteins were detected with an anti-His antibody. RNA polymerase (RNAP) was used as a loading control. *P < 0.05; **P < 0.01; ***P < 0.001.

According to the RNA-seq data, we found that the deletion of *luxS* promotes T6SS expression in *H*. *elongata* (Figure 7A and Supplementary Table S3). qRT-PCR analysis confirmed that the expression of T6SS-related genes *vgrG1, clpV, vipA*, and *vipB* was significantly increased in the Δ*luxS* mutant compared with the wild-type and complemented strains (*P* < 0.05; Figure 7B). Exogenous AI-2 supplementation restored T6SS gene expression to levels comparable to the wild-type in the Δ*luxS* mutant (Supplementary Figure S1C). Additionally, the loss of *luxS* significantly enhanced the promoter activity of T6SS in the Δ*luxS* mutant (*P* < 0.01; Figure 7C).

To further validate this finding, we assessed the secretion of Hemolysin co-regulated protein (Hcp), which is both a structural protein and a secretion protein of T6SS (Basler, 2015; Manera et al., 2021), in the wild-type, Δ*luxS* mutant, and complemented strains. In order to detect the secretion of Hcp, a plasmid expressing Hcp-His_6_ was introduced into indicated *H*. *elongata* strains via conjugation to produce the His-tagged Hcp. For the pellet fraction, RNA polymerase (RNAP) was used as a loading control. Western blotting analysis confirmed that Hcp secretion was elevated in Δ*luxS* mutant relative to the wild-type and complemented strains (Figure 7D). Collectively, these results suggest that the LuxS/AI-2 QS system downregulates T6SS in *H*. *elongata*.

## Discussion

Previous studies have shown that the LuxS/AI-2 QS system plays an essential role in bacterial adaptation and survival in their environment (Wang et al., 2018, 2019; Cao et al., 2023; Zhang et al., 2025). However, the roles of the LuxS/AI-2 QS system in halophiles are largely unknown. In this work, we report that the LuxS/AI-2 QS system regulates *doeCD* transcription to modulate intracellular ectoine levels, thus facilitating osmotic adaptation in *H*. *elongata*. Additionally, the LuxS/AI-2 QS system regulates motility and T6SS in *H*. *elongata*. Exogenous addition of AI-2 to the Δ*luxS* mutant rescued these phenotypes, confirming that they are specifically mediated by AI-2 signaling rather than metabolic disruption of the activated methyl cycle (Supplementary Figure S1).

*H*. *elongata* can tolerate salt concentrations well above 1.7 M NaCl, relying on the synthesis and accumulation of compatible solutes, especially ectoine (Kindzierski et al., 2017; Hermann et al., 2020; Hobmeier et al., 2022). Previous studies have demonstrated that the ectoine-deficient Δ*ectABC* mutant strain is unable to tolerate salt concentrations above 0.5 M NaCl (Goller et al., 1998; Grammann et al., 2002), emphasizing the critical role of ectoine for osmotic adaptation in *H*. *elongata*. Intriguingly, our results indicate that the AI-2 activity of *H*. *elongata* is closely related to salinity and the deletion of *luxS* impairs the growth of *H*. *elongata* at various salinities, suggesting the LuxS/AI-2 QS system contributes to osmotic adaptation in *H*. *elongata* (Figures 2, 3). The intracellular concentration of ectoine in *H*. *elongata* is mainly controlled by its synthesis and degradation. The transcript level of the *ectABC* operon is controlled by σ^70^ and σ^38^ promoters upstream of *ectA* and a σ^54^ promoter upstream of *ectC* (Schwibbert et al., 2011; Stiller et al., 2018). However, the transcript level and promoter activity of the *ectABC* operon are not affected by *luxS* deletion (Figures 5B, D). The genes *doeABCD* encodes four enzymes for degrading ectoine to aspartate (Hermann et al., 2020). DoeX regulates expression of the *doeAB* operon but not the *doeCD* genes (Schwibbert et al., 2011). Our data indicate that the LuxS/AI-2 QS system represses the transcription of *doeCD* genes, and the deletion of *luxS* decreases the intracellular ectoine levels (Figure 5). These findings reveal that *H*. *elongata* employs the LuxS/AI-2 QS system to fine-tune intracellular ectoine concentrations by repressing *doeCD* transcription, thereby achieving precise osmotic adaptation to fluctuating salinity. Although complementation was mediated by a constitutive promoter, confirming that *luxS* is required for the observed adaptations, whether the native salt-dependent transcriptional dynamics of *luxS* are physiologically necessary remains to be determined. Furthermore, the molecular mechanism by which the LuxS/AI-2 QS system represses *doeCD* transcription remains to be elucidated. Whether the downstream effector of the LuxS/AI-2 QS system directly or indirectly regulates the *doeCD* promoter remains unclear.

Motility is a key behavior enabling bacteria to find an optimal environment (Mitchell and Kogure, 2006; Moreno-Gamez, 2022). Previous studies have revealed diverse regulatory relationships between the LuxS/AI-2 QS system and motility (Pereira et al., 2013; Wang et al., 2018; Song and Wood, 2021; Zhang et al., 2025). In agreement with these reports, our findings show that the LuxS/AI-2 QS system enhances motility by upregulating nearly all flagellar-related genes in *H*. *elongata* (Figure 6). Notably, genes involved in flagellar assembly and function in *H*. *elongata* are closely associated with salinity (Hobmeier et al., 2022; Yu et al., 2022). These genes have higher transcription levels at high salt concentrations than at low salt concentrations (Hobmeier et al., 2022; Yu et al., 2022). Here, we show that the AI-2 activity of *H*. *elongata* exhibits similar trends at varying salt concentrations (Figure 2A), suggesting that *H*. *elongata* may employ the LuxS/AI-2 QS system to coordinate motility with salinity changes, enabling it to navigate toward optimal osmotic conditions.

T6SS is a widely distributed transmembrane macromolecular apparatus employed by many Gram-negative bacteria to translocate effectors into adjacent cells or the extracellular environment (Basler, 2015; Singh and Kumari, 2023). Traditionally, T6SS is recognized as an anti-bacterial weapon for interspecies competition (Allsopp and Bernal, 2023; Singh and Kumari, 2023). However, in some species, T6SS is also involved in combating diverse stresses (Lin et al., 2017; Si et al., 2017; Li et al., 2021; Lin et al., 2021; Yu et al., 2021). For example, in *Yersinia pseudotuberculosis*, OxyR, HpaR, and ZntR coordinately activate the expression of T6SS4 to acquire Zn^2+^ by secreting a Zn^2+^-binding effector YezP, thereby alleviating oxidative stresses (Wang et al., 2015a, 2017, 2020). A previous study showed that *H*. *elongata* T6SS is upregulated after 2.2 M NaCl (13%) shock treatment (Yu et al., 2024). In *Pseudomonas syringae* B728a, 10 genes in the T6SS are increased with the osmotic pressure increasing (Freeman et al., 2013). Intriguingly, we observed that the LuxS/AI-2 QS system represses T6SS expression and effector secretion in *H*. *elongata*. Whether the LuxS/AI-2 QS-mediated regulation of T6SS has any adaptive role in osmotic adaptation remains to be determined and is a subject for future investigation.

In summary, we revealed that the LuxS/AI-2 QS system regulates osmotic adaptation, motility, and T6SS in *H*. *elongata*. These findings shed light on the functional roles of the LuxS/AI-2 QS system in *H*. *elongata*, providing new insights into the regulatory mechanism underlying osmoadaptation in halophiles.

## Data Availability

The RNA-seq data from this study is available in the NCBI Sequence Read Archive (SRA) under the accession number PRJNA1427421.
