# Supplementary material for: The LuxS/AI-2 quorum sensing system regulates osmotic adaptation, motility, and type VI secretion system in Halomonas elongata
Source: Front Microbiol. 2026 Jun 23;17:1850263. doi: 10.3389/fmicb.2026.1850263 (PMC13337758; doi:10.3389/fmicb.2026.1850263)
Supplement: Supplementary file 1 [file Supplementary_file_1.docx]

**Table S1. Bacterial strains and plasmids used in this study.**

| Strains or plasmids | Relevant genotype description* | References |
| --- | --- | --- |
| ***E*. *coli*** |  |  |
| BL21(DE3) | Host for expression vector pET28a | Novagen |
| TG1 | Host for cloning | Novagen |
| S17-1 *λ*pir | *λ*-pir lysogen of S17-1, F *thi pro hsdR* [RP4-2 Tc::Mu Km::Tn7(Tp Sm)] | Laboratory  stock |
| ***H. elongata*** |  |  |
| *H*. *elongata* DSM 2581 | Wild-type | Laboratory  stock |
| Δ*luxS* | *luxS* gene deleted in *H. elongata* DSM 2581 | This study |
| ***V*. *harveyi*** |  |  |
| *V*. *harveyi* MM32 | *luxN*::*cat luxS*::Tn5*kan* | (Bassler et al., 1993) |
| **Plasmids** |  |  |
| pBBR1MCS1 | Expression vector contains a *lac* promoter, Cm^r^ | Laboratory  stock |
| pBBR1MCS1-*luxS* | pBBR1MCS1 carries *luxS* coding region at down-stream of the *lac* promoter, Cm^r^ | This study |
| pBBR1MCS2 | Expression vector contains a *lac* promoter, Km^r^ | Laboratory  stock |
| pBBR1MCS2-*hcp*-*his*_6_ | pBBR1MCS2 carries *hcp*-*his*_6_ coding region at down-stream of the *lac* promoter, Km^r^ | This study |
| pK18*mobsacB* | *sacB*-based suicide vector for gene knockout, Km^r^ | Laboratory  stock |
| pK18*mobsacB*-Δ*luxS* | Containing *luxS* knockout fragment, Km^r^ | This study |
| pET28a | Expression vector with N-terminal hexahistidine affinity tag, Km^r^ | Novagen |
| pET28a-*luxS* | pET28a carrying *luxS* coding region, Km^r^ | This study |
| pET28a-*pfs* | pET28a carrying *pfs* coding region, Km^r^ | This study |
| pKHGZ | Expression reporter plasmid carrying the promoterless *sfGFP* gene, Km^r^ | Laboratory  stock |
| P*luxS*-*sfGFP* | pKHGZ containing *luxS* promoter region, Km^r^ | This study |
| P*ectA*-*sfGFP* | pKHGZ containing *ectA* promoter region, Km^r^ | This study |
| P*doeC*-*sfGFP* | pKHGZ containing *doeC* promoter region, Km^r^ | This study |
| P*fliF*-*sfGFP* | pKHGZ containing *fliF* promoter region, Km^r^ | This study |
| P*t6ss*-*sfGFP* | pKHGZ containing *T6SS* promoter region, Km^r^ | This study |

*Cm^r^ and Km^r^ represent resistance to chloramphenicol and kanamycin at 20 and 50 μg/ml, respectively.

**Table S2. Primers used in this study.**

| **Primers** | **Sequence（5′ to 3′）** | **Function** |
| --- | --- | --- |
| *luxS*F-*Kpn*I | GGGAACAAAAGCTG**GGTACC**ATGAGCGACAAGATGAACGT | To generate pBBR1MCS1-*luxS* |
| *luxS*R-*Hind*III | AGGAATTCGATATC**AAGCTT**TCAGGCGAACACCTGGGTCC |  |
| *hcp*F-*Kpn*I | GGGAACAAAAGCTG**GGTACC**ATGCCAACCCCATGTTATA | To generate pBBR1MCS2-*hcp*-*his*_6_ |
| *hcp*R-*Hind*III | AGGAATTCGATATC**AAGCTT**TTAGTGGTGGTGGTGGTGGTGGCCTTCGATCGGGG |  |
| *luxS*UF-*BamH*I | AGCTCGGTACCCGG**GGATCC**CCCAACTGATCGATGGCAAG | To generate pK18*mobsacB*-Δ*luxS* |
| *luxS*UR | GGCTTCAGGCCACGGCACCTCGCGATTCGG |  |
| *luxS*DF | AGGTGCCGTGGCCTGAAGCCTGGTTGAAAA |  |
| *luxS*DR-*Sal*I | TTGCATGCCTGCAG**GTCGAC**AATGAACTAAAACGACACCG |  |
| *luxS*F-*BamH*I | CGC**GGATCC**ATGAGCGACAAGATGAACGT | To generate pET28a-*luxS* |
| *luxS*R-*Sal*I | ACGC**GTCGAC**TCAGGCGAACACCTGGGTCC |  |
| *pfs*F-*BamH*I | CGC**GGATCC**ATGAAACGCATCGGTATC | To generate pET28a-*pfs* |
| *pfs*F-*Sal*I | ACGC**GTCGAC**TCACGACTCGGCGGTGGC |  |
| P*luxS*F-*EcoR*I | CCCCCGGGCTGCAG**GAATTC**ATATCGTCGGCCGCCCCATG | To generate P*luxS*-*sfGFP* |
| P*luxS*R-*Sal*I | TCGCCTTTGCTCAT**GTCGAC**CACGGCACCTCGCGATTCGG |  |
| P*ectA*F-*EcoR*I | CCCCCGGGCTGCAG**GAATTC**CGACCTTCGGGCAATCCTGTC | To generate P*ectA*-*sfGFP* |
| P*ectA*R-*Sal*I | TCGCCTTTGCTCAT**GTCGAC**TGTCGTGGTTCGCTGTAG |  |
| P*doeC*F-*EcoR*I | CCCCCGGGCTGCAG**GAATTC**ACCTATCAGCGGCTGATCGA | To generate P*doeC*-*sfGFP* |
| P*doeC*R-*Sal*I | TCGCCTTTGCTCAT**GTCGAC**AAGCACCTCCCCTGACGCGC |  |
| P*fliF*F-*EcoR*I | CCCCCGGGCTGCAG**GAATTC**TGCGACTCCGGGGTCAGGAT | To generate P*fliF*-*sfGFP* |
| P*fliF*R-*Sal*I | TCGCCTTTGCTCAT**GTCGAC**GCGGGCCTCCCCGCTCGTCC |  |
| P*t6ss*F-*EcoR*I | CCCCCGGGCTGCAG**GAATTC**GCCAAGGTTGCGGTGTCG | To generate P*T6SS*-*sfGFP* |
| P*t6ss*R-*Sal*I | TCGCCTTTGCTCAT**GTCGAC**GGCGAACCTTCTCTCTGCAA |  |
| *luxS*-QF | GGCGACACCATCCACAAG | qRT-PCR |
| *luxS*-QR | CGCCGTCGTAGTCGTCAT |  |
| *doeC*-QF | ACGGCAAGTGGACTCACGG |  |
| *doeC*-QR | TCATACCAGGCCAGCAGACG |  |
| *doeD*-QF | TTCCACGCCTCCACCCA |  |
| *doeD*-QR | GCCTGCTGATAAATCGCCTC |  |
| *ectA*-QF | CCACAGAGCCCTTTACACCC |  |
| *ectA*-QR | GGAAACGAAGCCGACGAT |  |
| *ectB*-QF | GCGAGGGCGGTATCAATG |  |
| *ectB*-QR | GCGGAAGGTGCCGTTGT |  |
| *ectC*-QF | TGGCCGATGGCAAGATC |  |
| *ectC*-QR | GGTGCGTAGGAACCGTCTT |  |
| flhA-QF | TCGCCCAATGCCTATCTGTT |  |
| flhA-QR | GCTCGGTCCCGATCCATAC |  |
| flhB-QF | TAGCAGCAGCGACGAAGAAA |  |
| flhB-QR | CATCGGCGAGGTCTCCATT |  |
| fliF-QF | GTTTCGCCGACGAACTCCA |  |
| fliF-QR | GCGCACCTGTTTACCCATCT |  |
| fliF-QF | CGTCACGGTGCTCGATCAGA |  |
| fliF-QR | TAGCGTTCGGCGGTTTCC |  |
| flgG-QF | AGGGATTCTTCCAGGTTCGC |  |
| flgG-QR | CCGTCTTCGCCGATACTCA |  |
| flgF-QF | GGCGATGGCGAACTGGATA |  |
| flgF-QR | ACAGGCCGTCACCGTCTACC |  |
| vgrG1-QF | GGGGCGACTACGAACACTACGA |  |
| vgrG1-QR | TGGACCACCTTGACGACCTG |  |
| clpV-QF | GGGGTAGGCAAGACCGAGAC |  |
| clpV-QR | CGAGCAGCACCACCGAATAG |  |
| vipA-QF | CTTCTGGTGGTCGGTGATTT |  |
| vipA-QR | GTTCGCGGGCCTCATCTT |  |
| vipB-QF | GACAACGCCGCCTTCTTT |  |
| vipB-QR | CCTGATCTGCGACGTACTGG |  |
| 16S RNA-F | AGCGTGGGTAGCAAACAG |  |
| 16S RNA-R | GAAGGCACCAATCCATCTC |  |

Underlined sites indicate overlap fragments added for Seamless Cloning. Letters in boldface denote the restriction enzyme cutting sites.

**Table S3. Differentially expressed genes in Δ*luxS* mutant compared to the *H*. *elongata* wild-type detected by RNA-seq.**

| **Locus in**  ***H*. *elongata*** | **Gene name** | **Description** | **Fold change**  **(log2)** |
| --- | --- | --- | --- |
| HELO_1076 |  | spore coat U domain-containing protein | 1.20 |
| HELO_1077 |  | spore coat U domain-containing protein | 1.14 |
| HELO_1078 |  | molecular chaperone | 1.28 |
| HELO_1079 |  | fimbria/pilus outer membrane usher protein | 1.39 |
| HELO_1080 |  | spore coat U domain-containing protein | 1.13 |
| HELO_1099 |  | ammonium transporter | 1.26 |
| HELO_1100 |  | P-II family nitrogen regulator | 1.84 |
| HELO_1144 |  | alpha-hydroxy acid oxidase | 2.11 |
| HELO_1148 |  | TRAP transporter substrate-binding protein | 1.16 |
| HELO_1149 |  | TRAP transporter small permease | 1.58 |
| HELO_1150 |  | TRAP transporter large permease | 1.46 |
| HELO_1151 |  | mannitol dehydrogenase family protein | 1.52 |
| HELO_1152 | *uxuA* | mannonate dehydratase | 1.64 |
| HELO_1207 |  | sugar phosphate isomerase/epimerase | 2.71 |
| HELO_1208 |  | hydroxypyruvate isomerase family protein | 2.99 |
| HELO_1209 |  | GMC oxidoreductase | 2.79 |
| HELO_1210 |  | gluconate 2-dehydrogenase subunit 3 family protein | 3.03 |
| HELO_1211 |  | DUF1080 domain-containing protein | 2.99 |
| HELO_1293 |  | DEAD/DEAH box helicase | 1.16 |
| HELO_1321 |  | type IV pilin protein | 1.24 |
| HELO_1322 |  | prepilin-type N-terminal cleavage/methylation domain-containing protein | 2.28 |
| HELO_1323 |  | PilW family protein | 2.29 |
| HELO_1324 |  | hypothetical protein | 2.21 |
| HELO_1339 |  | hypothetical protein | 1.26 |
| HELO_1494 |  | YiiX/YebB-like N1pC/P60 family cysteine hydrolase | 2.21 |
| HELO_1495 |  | DUF4878 domain-containing protein | 2.17 |
| HELO_1533 |  | YeiH family protein | 1.07 |
| HELO_1541 |  | TraX family protein | 1.03 |
| HELO_1699 |  | sulfite exporter TauE/SafE family protein | 1.01 |
| HELO_1733 |  | SIMPL domain-containing protein | 1.03 |
| HELO_1814 |  | glucan biosynthesis protein | 1.71 |
| HELO_1870B |  | hypothetical protein | 1.25 |
| HELO_2046 |  | HlyD family secretion protein | 1.64 |
| HELO_2047 |  | peptidase domain-containing ABC transporter | 2.08 |
| HELO_2054 |  | sodium:calcium antiporter | 1.35 |
| HELO_2069 |  | hypothetical protein | 1.32 |
| HELO_2295 | *ehuB* | ectoine/hydroxyectoine ABC transporter substrate-binding protein | 1.06 |
| HELO_2313 |  | DUF3307 domain-containing protein | 1.12 |
| HELO_2396 | *rfbB* | dTDP-glucose 4,6-dehydratase | 1.30 |
| HELO_2397H |  | glycosyltransferase family 2 protein | 1.07 |
| HELO_2427 |  | CaiB/BaiF CoA-transferase family protein | 1.41 |
| HELO_2428 |  | DUF1513 domain-containing protein | 1.01 |
| HELO_2628 |  | linear amide C-N hydrolase | 1.27 |
| HELO_2667 |  | V-type ATP synthase subunit A | 1.21 |
| HELO_2668 |  | hypothetical protein | 1.45 |
| HELO_2669 |  | hypothetical protein | 1.54 |
| HELO_2670 |  | ATP synthase subunit C | 1.48 |
| HELO_2671 |  | V-type ATP synthase subunit I | 1.89 |
| HELO_2672 |  | V-type ATP synthase subunit D | 1.49 |
| HELO_2673 |  | V-type ATP synthase subunit B | 1.52 |
| HELO_2674 | *arcC* | carbamate kinase | 1.65 |
| HELO_2675 |  | ornithine carbamoyltransferase | 1.51 |
| HELO_2676 | *arcA* | arginine deiminase | 1.55 |
| HELO_2677 | *arcD* | arginine-ornithine antiporter | 1.42 |
| HELO_2678 |  | hypothetical protein | 1.78 |
| HELO_2679 |  | YqaE/Pmp3 family membrane protein | 2.05 |
| HELO_2681 |  | GlsB/YeaQ/YmgE family stress response membrane protein | 2.39 |
| HELO_2813 |  | Hint domain-containing protein | 1.30 |
| HELO_2846 |  | amino acid adenylation domain-containing protein | 3.55 |
| HELO_2860 |  | hypothetical protein | 1.70 |
| HELO_2871 |  | helix-hairpin-helix domain-containing protein | 1.44 |
| HELO_2893 | *hmpA* | NO-inducible flavohemoprotein | 1.14 |
| HELO_2905 |  | hypothetical protein | 1.50 |
| HELO_2906 |  | DUF4123 domain-containing protein | 1.77 |
| HELO_2907 | *vgrG1* | type VI secretion system Vgr family protein | 2.07 |
| HELO_2908 | *PAAR* | type VI secretion system PAAR protein | 1.76 |
| HELO_2909 |  | GNAT family N-acetyltransferase | 2.14 |
| HELO_2910 | *vasK* | type VI secretion system membrane subunit | 1.65 |
| HELO_2911 | *vasJ* | type VI secretion system protein | 1.82 |
| HELO_2912 | *vasI* | type VI secretion system-associated protein | 2.01 |
| HELO_2913 | *vasH* | sigma-54-dependent Fis family transcriptional regulator | 1.78 |
| HELO_2914 | *clpV* | type VI secretion system ATPase | 1.69 |
| HELO_2915 | *vasF* | type IVB secretion system protein IcmH/DotU | 1.65 |
| HELO_2916 | *vasE* | type VI secretion system baseplate subunit | 1.62 |
| HELO_2917 | *vasD* | type VI secretion system lipoprotein | 1.27 |
| HELO_2918 | *vasC* | type VI secretion system-associated FHA domain protein | 1.35 |
| HELO_2919 | *vasB* | type VI secretion system baseplate subunit | 1.51 |
| HELO_2920 | *vasA* | type VI secretion system baseplate subunit | 1.66 |
| HELO_2921 |  | type VI secretion system baseplate subunit | 1.78 |
| HELO_2922 | *vipB* | type VI secretion system contractile sheath large subunit | 1.34 |
| HELO_2923 | *vipA* | type VI secretion system contractile sheath small subunit | 1.28 |
| HELO_2924 |  | hypothetical protein | 1.26 |
| HELO_2926 |  | hypothetical protein | 1.25 |
| HELO_2927 |  | DUF4123 domain-containing protein | 1.59 |
| HELO_2928 | *vgrG2* | type VI secretion system Vgr family protein | 1.96 |
| HELO_2929 | *hcp1* | Hcp family type VI secretion system effector | 1.67 |
| HELO_3009 |  | endonuclease/exonuclease/phosphatase family protein | 1.16 |
| HELO_3024 |  | DUF1800 family protein | 1.15 |
| HELO_3025 |  | DUF2501 domain-containing protein | 3.46 |
| HELO_3083 |  | GlsB/YeaQ/YmgE family stress response membrane protein | 1.21 |
| HELO_3085 |  | solute:sodium symporter family transporter | 1.92 |
| HELO_3125 |  | hypothetical protein | 1.10 |
| HELO_3150 |  | Yip1 family protein | 1.20 |
| HELO_3183 |  | DUF2182 domain-containing protein | 1.05 |
| HELO_3204 |  | Gfo/Idh/MocA family protein | 2.03 |
| HELO_3205 |  | sugar phosphate isomerase/epimerase | 2.36 |
| HELO_3206 |  | nucleoside permease | 2.30 |
| HELO_3209 |  | murein L,D-transpeptidase | 1.14 |
| HELO_3281 |  | SulP family inorganic anion transporter | 2.98 |
| HELO_3343 |  | PRC-barrel domain-containing protein | 2.26 |
| HELO_3418 |  | AEC family transporter | 2.11 |
| HELO_3552 |  | fumarylacetoacetate hydrolase family protein | 1.00 |
| HELO_3553 |  | zinc-binding alcohol dehydrogenase family protein | 1.33 |
| HELO_3554 |  | UxaA family hydrolase | 1.30 |
| HELO_3558 |  | TRAP transporter large permease | 1.08 |
| HELO_3636 | *pgl* | 6-phosphogluconolactonase | 1.02 |
| HELO_3637 | *zwf* | glucose-6-phosphate dehydrogenase | 1.01 |
| HELO_3647 |  | nodulation protein NfeD | 1.05 |
| HELO_3660 | *eutB* | hydroxyectoine utilization dehydratase EutB | 1.08 |
| HELO_3661 | *doeD* | aspartate aminotransferase family protein | 1.12 |
| HELO_3662 | *doeC* | NAD-dependent succinate-semialdehyde dehydrogenase | 1.08 |
| HELO_3675 |  | carbohydrate ABC transporter permease | 1.17 |
| HELO_3679 | *ugpC* | sn-glycerol-3-phosphate ABC transporter ATP-binding protein UgpC | 1.08 |
| HELO_3686 | *malT* | HTH-type transcriptional regulator MalT | 1.76 |
| HELO_3687 |  | alpha-amylase family glycosyl hydrolase | 1.10 |
| HELO_3756 |  | type IV pilus secretin PilQ | 1.16 |
| HELO_3895 |  | LysR family transcriptional regulator | 1.05 |
| HELO_3896 |  | low temperature requirement protein A | 1.11 |
| HELO_3909 |  | CocE/NonD family hydrolase | 1.06 |
| HELO_4022 | *dnaB* | replicative DNA helicase | 2.97 |
| HELO_4023 |  | hypothetical protein | 4.46 |
| HELO_4024 |  | hypothetical protein | 3.30 |
| HELO_4025 |  | hypothetical protein | 3.02 |
| HELO_4026 |  | hypothetical protein | 3.15 |
| HELO_4027 |  | hypothetical protein | 3.86 |
| HELO_4028 |  | conjugal transfer nickase/helicase domain-containing protein | 2.63 |
| HELO_4029 |  | DUF2786 domain-containing protein | 2.63 |
| HELO_4030 |  | hypothetical protein | 2.40 |
| HELO_4031 |  | ParB family protein | 2.20 |
| HELO_4032 |  | DUF2857 domain-containing protein | 2.44 |
| HELO_4033 |  | STY4528 family pathogenicity island replication protein | 1.63 |
| HELO_4034 |  | PFL_4669 family integrating conjugative element protein | 2.00 |
| HELO_4035 |  | DUF3158 family protein | 1.76 |
| HELO_4036 |  | hypothetical protein | 1.92 |
| HELO_4036A |  | hypothetical protein | 3.02 |
| HELO_4037 |  | single-stranded DNA-binding protein | 2.78 |
| HELO_4038 |  | PH domain-containing protein | 2.20 |
| HELO_4039 |  | hypothetical protein | 1.69 |
| HELO_4039A |  | TraR/DksA C4-type zinc finger protein | 1.19 |
| HELO_4040 |  | DNA topoisomerase III | 1.35 |
| HELO_4041 |  | hypothetical protein | 1.51 |
| HELO_4042 |  | type IV pili sensor histidine kinase/response regulator | 3.50 |
| HELO_4043 |  | hypothetical protein | 4.20 |
| HELO_4044 |  | hypothetical protein | 3.55 |
| HELO_4045 |  | TIGR03759 family integrating conjugative element protein | 3.50 |
| HELO_4046 |  | transglycosylase SLT domain-containing protein | 3.35 |
| HELO_4047 |  | integrating conjugative element protein | 3.57 |
| HELO_4048 |  | hypothetical protein | 4.25 |
| HELO_4049 |  | PcfJ domain-containing protein | 3.02 |
| HELO_4050 | *traD* | type IV conjugative transfer system coupling protein TraD | 2.95 |
| HELO_4051 |  | TIGR03747 family integrating conjugative element membrane protein | 2.49 |
| HELO_4052 |  | RAQPRD family integrative conjugative element protein | 3.33 |
| HELO_4053 |  | TIGR03758 family integrating conjugative element protein | 3.03 |
| HELO_4054 |  | TIGR03745 family integrating conjugative element membrane protein | 2.92 |
| HELO_4055 |  | TIGR03750 family conjugal transfer protein | 3.78 |
| HELO_4056 |  | PFL_4703 family integrating conjugative element protein | 3.79 |
| HELO_4057 |  | TIGR03749 family integrating conjugative element protein | 3.07 |
| HELO_4058 |  | TIGR03752 family integrating conjugative element protein | 2.89 |
| HELO_4059 |  | TIGR03751 family conjugal transfer lipoprotein | 2.79 |
| HELO_4060 |  | conjugative transfer ATPase | 1.95 |
| HELO_4061 |  | hypothetical protein | 1.37 |
| HELO_4062 |  | hypothetical protein | 1.53 |
| HELO_4063 |  | DUF2726 domain-containing protein | 1.64 |
| HELO_4064 |  | DsbC family protein | 1.92 |
| HELO_4065 |  | hypothetical protein | 1.32 |
| HELO_4067 |  | TIGR03757 family integrating conjugative element protein | 2.34 |
| HELO_4068 |  | TIGR03756 family integrating conjugative element protein | 1.94 |
| HELO_4069 |  | hypothetical protein | 1.18 |
| HELO_4070 |  | hypothetical protein | 1.28 |
| HELO_4076B |  | DUF4123 domain-containing protein | 1.52 |
| HELO_4077 | *vgrG3* | type VI secretion system Vgr family protein | 1.93 |
| HELO_4078 | *hcp2* | Hcp family type VI secretion system effector | 2.41 |
| HELO_4080A |  | hypothetical protein | 1.50 |
| HELO_4081 |  | STY4534 family ICE replication protein | 3.72 |
| HELO_4089 |  | hypothetical protein | 3.77 |
| HELO_4089A |  | hypothetical protein | 3.52 |
| HELO_4090 |  | hypothetical protein | 2.13 |
| HELO_4090A |  | hypothetical protein | 4.08 |
| HELO_4091 |  | ArdC family protein | 2.84 |
| HELO_4094 | *mobH* | MobH family relaxase | 2.04 |
| HELO_4095 |  | site-specific integrase | 1.42 |
| HELO_4098A |  | alpha/beta fold hydrolase | 3.31 |
| HELO_4099 |  | cytochrome P450 | 2.57 |
| HELO_4113 |  | type IV pilus twitching motility protein PilT | 1.23 |
| HELO_4171 |  | hypothetical protein | 1.32 |
| HELO_4242 | *gap* | type I glyceraldehyde-3-phosphate dehydrogenase | 1.61 |
| HELO_4290 |  | WYL domain-containing protein | 1.12 |
| HELO_4384 |  | EAL domain-containing protein | 1.32 |
| HELO_4385 |  | hypothetical protein | 3.84 |
| HELO_1035 |  | TRAP transporter substrate-binding protein | -1.46 |
| HELO_1036 |  | TRAP transporter small permease | -1.12 |
| HELO_1051 |  | TRAP transporter substrate-binding protein | -1.05 |
| HELO_1135 |  | tripartite tricarboxylate transporter substrate binding protein | -1.10 |
| HELO_1137 |  | tripartite tricarboxylate transporter permease | -1.01 |
| HELO_1167 |  | TRAP transporter large permease | -1.34 |
| HELO_1168 |  | TRAP transporter small permease | -1.23 |
| HELO_1169 |  | TRAP transporter substrate-binding protein | -1.49 |
| HELO_1170 |  | D-glycerate dehydrogenase | -1.12 |
| HELO_1195 |  | 6,7-dimethyl-8-ribityllumazine synthase | -1.02 |
| HELO_1295 |  | cytosine permease | -1.79 |
| HELO_1296 |  | 5-guanidino-2-oxopentanoate decarboxylase | -1.14 |
| HELO_1297 |  | YjiH family protein | -1.56 |
| HELO_1434 | *aqpZ* | aquaporin Z | -1.00 |
| HELO_1501 |  | malonyl-CoA decarboxylase | -1.00 |
| HELO_1502 |  | TRAP transporter large permease | -1.90 |
| HELO_1503 |  | TRAP transporter small permease subunit | -1.38 |
| HELO_1504 | *dctP* | TRAP transporter substrate-binding protein DctP | -1.92 |
| HELO_1548 |  | ABC transporter substrate-binding protein | -1.35 |
| HELO_1549 |  | ABC transporter permease | -1.26 |
| HELO_1553 |  | SMP-30/gluconolactonase/LRE family protein | -1.45 |
| HELO_1558 | *dctP* | TRAP transporter substrate-binding protein DctP | -1.53 |
| HELO_1558A |  | TRAP transporter small permease subunit | -1.05 |
| HELO_1559 |  | TRAP transporter large permease subunit | -1.15 |
| HELO_1584 |  | heme-binding protein | -1.63 |
| HELO_1585 | *glcF* | glycolate oxidase subunit GlcF | -1.20 |
| HELO_1586 | *glcE* | glycolate oxidase subunit GlcE | -1.07 |
| HELO_1598 |  | YfcC family protein | -1.31 |
| HELO_1623 |  | tripartite tricarboxylate transporter permease | -1.22 |
| HELO_1624 |  | tripartite tricarboxylate transporter TctB family protein | -2.25 |
| HELO_1625 |  | tripartite tricarboxylate transporter substrate binding protein | -2.41 |
| HELO_1660 |  | YadA family autotransporter adhesin | -1.18 |
| HELO_1740 | *prpB* | methylisocitrate lyase | -1.33 |
| HELO_1741 | *prpC* | 2-methylcitrate synthase | -1.58 |
| HELO_1742 | *prpD* | 2-methylcitrate dehydratase | -1.55 |
| HELO_1901 |  | Na+/H+ antiporter NhaC family protein | -1.24 |
| HELO_2142 |  | AMP-binding protein | -1.08 |
| HELO_2143 |  | SDR family NAD(P)-dependent oxidoreductase | -1.11 |
| HELO_2343 | *luxS* | S-ribosylhomocysteine lyase | -21.66 |
| HELO_2421 |  | AMP-binding protein | -1.17 |
| HELO_2468 |  | NCS2 family permease | -1.48 |
| HELO_2483 |  | sugar ABC transporter substrate-binding protein | -1.10 |
| HELO_2484 |  | carbohydrate ABC transporter permease | -1.28 |
| HELO_2485 |  | carbohydrate ABC transporter permease | -1.18 |
| HELO_2486 |  | ABC transporter ATP-binding protein | -1.05 |
| HELO_2580 |  | LysR family transcriptional regulator | -1.28 |
| HELO_2581 |  | ABC transporter ATP-binding protein | -1.52 |
| HELO_2582 |  | ABC transporter ATP-binding protein | -1.59 |
| HELO_2583 |  | ABC transporter permease | -1.46 |
| HELO_2584 |  | ABC transporter permease | -1.50 |
| HELO_2585 |  | ABC transporter substrate-binding protein | -1.71 |
| HELO_2644 |  | hypothetical protein | -1.92 |
| HELO_2686 |  | propionyl-CoA synthetase | -1.09 |
| HELO_2786 | *araG* | L-arabinose ABC transporter ATP-binding protein AraG | -1.31 |
| HELO_2787 |  | arabinose ABC transporter substrate-binding protein | -1.06 |
| HELO_2790 |  | extracellular solute-binding protein | -1.03 |
| HELO_2798 |  | family 43 glycosylhydrolase | -1.60 |
| HELO_2801 |  | TRAP transporter substrate-binding protein | -1.72 |
| HELO_2804 |  | MFS transporter | -1.38 |
| HELO_2805 |  | family 43 glycosylhydrolase | -1.28 |
| HELO_2817 |  | aldehyde dehydrogenase family protein | -1.19 |
| HELO_2861 |  | Hint domain-containing protein | -1.35 |
| HELO_2936 |  | C4-dicarboxylate TRAP transporter substrate-binding protein | -1.10 |
| HELO_2962A |  | oligogalacturonate-specific porin KdgM family protein | -1.66 |
| HELO_2964A |  | TRAP transporter substrate-binding protein | -1.36 |
| HELO_2989 |  | ABC transporter ATP-binding protein | -1.42 |
| HELO_2990 |  | ABC transporter ATP-binding protein | -1.47 |
| HELO_3005 | *phnD* | phosphate/phosphite/phosphonate ABC transporter substrate-binding protein | -1.01 |
| HELO_3006 | *phnC* | phosphonate ABC transporter ATP-binding protein | -1.21 |
| HELO_3007 | *phnE* | phosphonate ABC transporter, permease protein PhnE | -1.45 |
| HELO_3070 |  | isocitrate lyase | -1.90 |
| HELO_3071 |  | hypothetical protein | -1.14 |
| HELO_3090 |  | YbiU family protein | -1.86 |
| HELO_3091 |  | ABC transporter ATP-binding protein | -1.92 |
| HELO_3092 |  | carbohydrate ABC transporter permease | -1.88 |
| HELO_3093 |  | carbohydrate ABC transporter permease | -1.35 |
| HELO_3094 |  | ABC transporter substrate-binding protein | -1.07 |
| HELO_3099 | *nagE* | N-acetylglucosamine-specific PTS transporter subunit IIBC | -1.00 |
| HELO_3258 |  | DUF3311 domain-containing protein | -1.55 |
| HELO_3282 |  | TRAP transporter substrate-binding protein | -1.31 |
| HELO_3291E |  | DOPA 4,5-dioxygenase family protein | -1.10 |
| HELO_3410 |  | TRAP transporter substrate-binding protein | -1.08 |
| HELO_3494 |  | sugar ABC transporter ATP-binding protein | -1.07 |
| HELO_3545 |  | 3'-5' exonuclease | -1.32 |
| HELO_3546 |  | putative nucleotidyltransferase substrate binding domain-containing protein | -1.08 |
| HELO_3547 |  | DUF4212 domain-containing protein | -1.58 |
| HELO_3548 |  | sodium:solute symporter family protein | -2.11 |
| HELO_3549 |  | hypothetical protein | -1.65 |
| HELO_3709 |  | outer membrane protein OmpK | -1.13 |
| HELO_3846 |  | peptide ABC transporter substrate-binding protein | -1.23 |
| HELO_3847 | *oppB* | oligopeptide ABC transporter permease OppB | -1.12 |
| HELO_3848 |  | ABC transporter permease | -1.11 |
| HELO_3849 |  | ABC transporter ATP-binding protein | -1.00 |
| HELO_3850 |  | porin | -1.79 |
| HELO_3931 |  | MBL fold metallo-hydrolase | -1.35 |
| HELO_3940 |  | aromatic ring-hydroxylating dioxygenase subunit alpha | -1.14 |
| HELO_3941 |  | PDR/VanB family oxidoreductase | -1.69 |
| HELO_3942 |  | TRAP transporter substrate-binding protein | -2.19 |
| HELO_3987 |  | short-chain fatty acid transporter | -1.09 |
| HELO_3996 | *dctP* | TRAP transporter substrate-binding protein DctP | -1.39 |
| HELO_3997 |  | TRAP transporter small permease subunit | -1.17 |
| HELO_3998 |  | TRAP transporter large permease subunit | -1.18 |
| HELO_4215 | *aldA* | aldehyde dehydrogenase | -1.19 |
| HELO_4216 |  | cytosine permease | -1.00 |
| HELO_4237 |  | sugar ABC transporter substrate-binding protein | -1.42 |
| HELO_4268 |  | TRAP transporter substrate-binding protein | -1.51 |
| HELO_4331 | *fliA* | RNA polymerase sigma factor | -2.17 |
| HELO_4332 | *flhE* | flagellar protein | -2.19 |
| HELO_4333 | *flhA* | flagellar biosynthesis protein | -2.72 |
| HELO_4334 | *flhB* | flagellar biosynthesis protein | -2.66 |
| HELO_4335 | *cheZ* | protein phosphatase | -1.74 |
| HELO_4335A | *cheY* | chemotaxis response regulator | -2.44 |
| HELO_4336 | *cheB* | chemotaxis response regulator protein-glutamate methylesterase | -1.91 |
| HELO_4337 | *cheR* | CheR family methyltransferase | -2.16 |
| HELO_4338 |  | methyl-accepting chemotaxis protein | -2.17 |
| HELO_4339 |  | methyl-accepting chemotaxis protein | -2.22 |
| HELO_4340 |  | methyl-accepting chemotaxis protein | -2.39 |
| HELO_4341 | *cheW* | chemotaxis protein | -2.43 |
| HELO_4342 | *cheA* | chemotaxis protein | -2.16 |
| HELO_4343 | *motB* | flagellar motor protein | -2.06 |
| HELO_4344 | *motA* | flagellar motor stator protein | -3.01 |
| HELO_4345 | *flhC* | flagellar transcriptional regulator | -1.51 |
| HELO_4345A | *flhD* | flagellar transcriptional regulator | -1.69 |
| HELO_4346 |  | EscU/YscU/HrcU family type III secretion system export apparatus switch protein | -1.46 |
| HELO_4347 | *fliK* | flagellar hook-length control protein | -2.04 |
| HELO_4348 | *fliT* | flagellar protein | -1.38 |
| HELO_4349 | *fliS* | flagellar export chaperone | -2.11 |
| HELO_4350 |  | DUF2835 domain-containing protein | -1.68 |
| HELO_4351 |  | tetratricopeptide repeat protein | -1.96 |
| HELO_4352 |  | flagellin | -1.83 |
| HELO_4353 |  | flagellin | -1.82 |
| HELO_4354 | *fliD1* | flagellar filament capping protein | -2.63 |
| HELO_4355 | *fliD2* | flagellar filament capping protein | -2.14 |
| HELO_4356 |  | methyl-accepting chemotaxis protein | -2.30 |
| HELO_4357 | *flaG* | flagellar protein | -2.18 |
| HELO_4358 | *fliE* | flagellar hook-basal body complex protein | -1.89 |
| HELO_4359 | *fliF* | flagellar basal-body MS-ring/collar protein | -3.00 |
| HELO_4360 | *fliG* | flagellar motor switch protein | -3.15 |
| HELO_4361 | *fliH* | flagellar assembly protein | -3.31 |
| HELO_4362 | *fliI* | flagellar protein export ATPase | -3.17 |
| HELO_4363 | *fliJ* | flagellar export protein | -3.48 |
| HELO_4364 | *fliK* | flagellar hook-length control protein | -2.88 |
| HELO_4365 | *fliL* | flagellar basal body-associated protein | -2.82 |
| HELO_4366 | *fliM* | flagellar motor switch protein | -2.85 |
| HELO_4366A | *fliN* | flagellar motor switch protein | -3.06 |
| HELO_4367 | *fliO* | flagellar biosynthetic protein | -3.12 |
| HELO_4368 | *fliP* | flagellar type III secretion system pore protein | -3.11 |
| HELO_4369 | *fliQ* | flagellar biosynthesis protein | -3.44 |
| HELO_4370 | *fliR* | flagellar biosynthetic protein | -2.48 |
| HELO_4371 | *flgL* | flagellar hook-associated protein | -2.07 |
| HELO_4372 | *flgK* | flagellar hook-associated protein | -2.11 |
| HELO_4373 | *flgJ* | flagellar assembly peptidoglycan hydrolase | -2.90 |
| HELO_4374 | *flgI* | flagellar basal body P-ring protein | -2.46 |
| HELO_4375 | *flgH* | flagellar basal body L-ring protein | -2.89 |
| HELO_4376 | *flgG* | flagellar basal-body rod protein | -3.48 |
| HELO_4377 | *flgF* | flagellar basal-body rod protein | -3.26 |
| HELO_4378 | *flgE* | flagellar hook protein | -3.51 |
| HELO_4379 | *flgD* | flagellar hook assembly protein | -3.35 |
| HELO_4380 | *flgC* | flagellar basal body rod protein | -3.28 |
| HELO_4381 | *flgB* | flagellar basal body rod protein | -3.02 |
| HELO_4382 | *flgA* | flagellar basal body P-ring formation chaperone | -2.61 |
| HELO_4382A | *flgM* | flagellar biosynthesis anti-sigma factor | -1.09 |
| HELO_4445 | *atpG* | F0F1 ATP synthase subunit gamma | -1.10 |


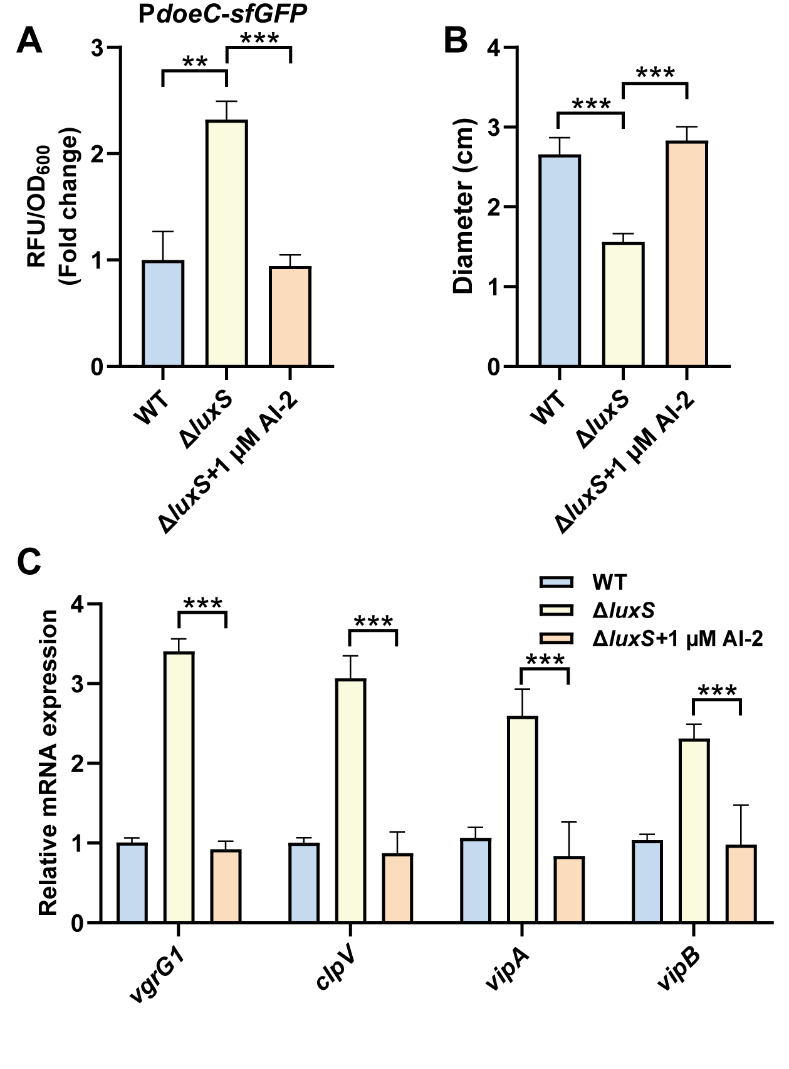


**Fig. S1. Exogenous AI-2 rescues Δ*luxS* mutant phenotypes.**

Exogenous AI-2 was added to the culture medium of the Δ*luxS* mutant. **A**. Promoter activity of *doeC*. Data are shown as mean ± SD from three independent biological replicates (n=3). Statistical significance was determined using one-way ANOVA with Tukey's post-hoc test. **B**. Swimming motility. Data are shown as mean ± SD from three independent biological replicates (n=3). Statistical significance was determined using one-way ANOVA with Tukey's post-hoc test. **C**. Relative mRNA levels of T6SS genes determined by qRT-PCR. Data are shown as mean ± SD from three independent biological replicates (n=3). Statistical significance was determined using two-way ANOVA with Tukey's post-hoc test. ** *P* < 0.01; *** *P* < 0.001.

**References**

Bassler, B.L., Wright, M., Showalter, R.E., and Silverman, M.R. (1993). Intercellular signalling in Vibrio harveyi: sequence and function of genes regulating expression of luminescence. *Mol Microbiol* 9(4)**,** 773-786. doi: 10.1111/j.1365-2958.1993.tb01737.x.
